# Modelling temperature effects on milk production: a study on Holstein cows at a Japanese farm

**DOI:** 10.1186/2193-1801-3-129

**Published:** 2014-03-07

**Authors:** Machiko Yano, Hideyasu Shimadzu, Toshiki Endo

**Affiliations:** Jiyu Gakuen College, 1-8-15 Gakuen-cho, Higashikurume-shi, Tokyo, 203-8521 Japan; School of Biology, Centre for Biological Diversity and Scottish Oceans Institute, University of St Andrews, Dyers Brae House, St Andrews, Fife KY16 9TH UK; Department of Mathematics, Keio University, 3-14-1 Hiyoshi Kohoku, Yokohama, 223-8522 Japan

**Keywords:** Milk production, Milk fat, Heat stress, Lactation curves, Modelling, Test-day data

## Abstract

Milk yield and its composition vary according to individual cows as well as to a variety of different environment conditions, such as temperature. Previous studies suggest that heat exerts considerable negative effects on milk production and its composition, especially during summer months. We investigate the production and fat composition of milk from individual dairy cows and develop a modelling framework that investigates the effect of temperature by extending a traditional lactation curve model onto a more flexible statistical modelling framework, a generalised additive model (GAM). The GAM simultaneously copes with multiple different conditions (temperature, parity, days of lactation, etc.), and, importantly, their non-linear relationships. Our analysis of retrospective data suggests that individual cows respond differently to heat; cows producing relatively high quantities of milk tend to be particularly sensitive to heat. Our model also suggests that most dairy cows studied fall into three distinct cases that underpin the variation of the milk fat ratio by different mechanisms.

**Electronic supplementary material:**

The online version of this article (doi:10.1186/2193-1801-3-129) contains supplementary material, which is available to authorized users.

## Background

It has been well recognised that milk yield and its composition vary according to individual cows as well as to a variety of different environment conditions, such as temperature. Previous studies indicate, for example, that heat exerts considerable negative effects on milk production. Extensive efforts have been made to quantify the effect of heat on milk production, investigating such factors as humidity, wind speed, daylight length, and temperature and humidity indices (THIs). The results generally suggest that heat stress results in decreased milk production (Barash et al. 2001; Bouraoui et al. 2002; West et al. 2003; Bohmanova et al. 2007) and altered composition (Bandaranayaka and Holmes 1976; McDowell et al. 1976; Schneider et al. 1988); since dairy cows prefer a relatively cool atmosphere, these findings are logical.

To investigate the extent to which the variation of milk production and its composition are driven by individual differences as well as differing environment conditions, including temperature effects, a number of modelling attempts have been undertaken. There are two major modelling streams: lactation curve models (Wood 1976) and random regression test-day models (Schaeffer 2004). The most challenging aspect of modelling is constructing a flexible model that copes with the non-linear nature of milk production and the individual differences in dairy cows; the actual functional relationships are far more complex than simple linear relationships. In this respect, the lactation curve model is a non-linear model, but it is not flexible enough to deal simultaneously with individual differences and other multiple differing conditions. On the other hand, the random regression test-day model is capable of describing both individual differences and other multiple conditions, but it often restricts its attention to particular linear relationships.

In this study, we aim to develop a flexible modelling framework that utilises the previous two modelling approaches. Our modelling framework is built directly on the lactation curve model. We extend this traditional model onto a well-known statistical modelling framework: the generalised additive model (GAM; Hastie and Tibshirani 1990). The GAM provides enhanced modelling flexibility that copes with both multiple differing conditions and individual differences, and is therefore effective in modelling non-linear relationships.

We model the effect of temperature on the yield and fat composition of milk produced by individual cows. Our analysis of retrospective data suggests that cows producing high quantities of milk are sensitive to heat and tend to decrease their milk production as the ambient temperature increases. Additionally, most dairy cows studied here fall into three distinct cases that underpin the variation of milk fat ratios by different mechanisms.

## Results

### Models

The composition of milk varies according to individual cows as well as to different environment conditions. We investigate two major components of milk production: (i) the milk yield, *y*, and (ii) the milk fat ratio, *z*^′^, as recorded in the test-day data (see Materials and methods). To investigate the extent to which the variation of these components is driven by different factors, a number of modelling attempts has been undertaken. These have utilised lactation curves (Allore et al. 1997; Barash et al. 2001; Bouraoui et al. 2002; Wood 1976) and test-day data modelling (Bignardi et al. 2012; Kettunen et al. 2000; Schaeffer 2004), independently fitting a single model to each component. In doing so, however, these studies have incorrectly made a model assumption of the error structure, which may lead to biased inference. We can clearly see this from the definition of the milk fat ratio:1

where *z* is the amount of milk fat. Here, the milk fat ratio, *z*^′^, is a function of the milk yield, *y*, and the milk fat yield, *z*. Accordingly, the variation of the milk fat ratio originates from that of the milk yield as well as the milk fat yield. In other words, the milk fat ratio is derived from the milk yield or the milk fat; they are, therefore, always dependent.

We here propose a simple modelling approach that properly copes with relationship (1). Our model is also related with traditional lactation curve models as well as random regression test-day models (see Discussion). We model the milk yield, *y*_*it*_, and the milk fat yield, *z*_*it*_, (not the milk fat ratio) from the *i*-th cow at time *t* in the natural logarithmic scale as23

where *ε*_*it*_ and *ξ*_*it*_ are respectively independent Gaussian noise with variance  and  between cows, *i*. The functions here, *s*_*j*_(·) and *t*_*j*_(·), are smoothing spline functions whose functional form can differ among the covariates, *x*_*j*_’s such as parity, days of lactation, calving month, amount of concentrate feed, and day length: the various calving conditions. Some of these can be individual-dependent, for which the notation should be *x*_*ijt*_, but we drop the subscript *i* for simplification.

The model here assumes a linear relationship with the daily maximum temperature, *w*_*t*_. This can be regarded as a linear approximation of the smooth non-linear function *s*(*w*_*t*_) or *t*(*w*_*t*_). Such an approximation is able to capture the temperature effect in a parsimonious way; the effect is now expressed by only one parameter, the temperature coefficient *a*_*i*_ or *b*_*i*_ that varies among individual cows, *i*. A negative value indicates decreased milk or milk fat production as the maximum temperature increases; a positive value indicates the opposite situation, increased milk or milk fat production, because of an increase of the maximum temperature.

The parameters *α*_*i*_, *a*_*i*_,  and the smooth function *s*_*j*_ in Equation (2) are estimable from the data under the generalised additive modelling (GAM; Wood 2006) framework (see Materials and methods). In contrast, the parameters *β*_*i*_, *b*_*i*_,  and the smooth function *t*_*j*_ in Equation (3) cannot be directly estimated from our test-day data since no records of milk fat, *z*_*it*_, are actually available. However, by noting the relationship (Equation (1)), they can be estimated through the milk fat ratio, *z*^′^, recorded in the test-day data. Since we know relationship (1), the milk fat ratio in the natural logarithmic scale can be described as4

where log(*y*_*it*_) is an offset term and *ξ*_*it*_ is an error term. We can then fit the models (Equations (2) and (3)) using the relationship given in Equation (4). A disregard for the offset term when fitting the model is equivalent to fitting a single independent model to the milk fat ratio. In doing so, if models (2) and (3) are correct, an inappropriate error structure is introduced, by minimising the sum of squared residuals . As Equation (4) shows, the correct procedure in parameter estimation should be to minimise , instead.

### Temperature effects on individual dairy cows

**Figure 1:**
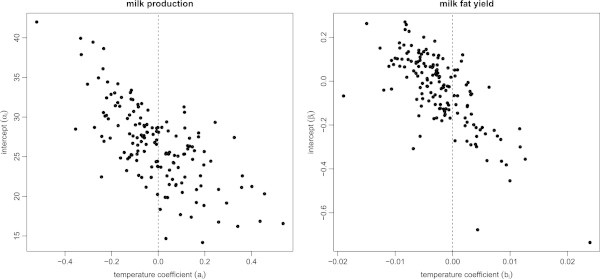
**Plots of the constants for milk production and the milk fat yield against the respective temperature coefficients.** The superposed dashed line separates individual cows into groups according to whether their temperature coefficient is positive or negative. This negative correlation between the constant and the temperature coefficient suggests that relatively highly productive cows are sensitive to heat.

Temperature has a greater influence on cows that produce relatively high amounts of milk and fat content. Figure 1 shows the scatter plots of the intercept of milk production, *α*_*i*_, (left) and the milk fat yield, *β*_*i*_, (right) against their respective temperature coefficient, *a*_*i*_ or *b*_*i*_. A negative temperature coefficient indicates decreased milk or milk fat production as the maximum temperature increases. A positive one implies increased milk or milk fat production due to an increase in the maximum temperature. Each plot shows a clear negative correlation, indicating that the cows that are relatively highly productive tend to be more sensitive to heat and may decrease their productivity when the temperature increases. Our results support the findings of previous studies (Johnston 1958; Bianca 1965; Barash et al. 2001) as well. They report that highly productive cows tend to have a relatively high body temperature, and are therefore more sensitive to heat.

### Variation of the milk fat ratio according to temperature

**Figure 2:**
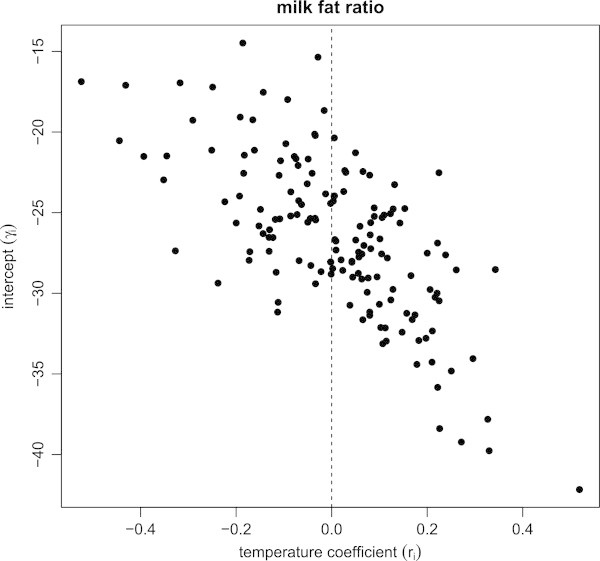
**Plot of the constant for the milk fat ratio against the temperature coefficient.** The superposed dashed line separates individual cows into groups according to whether their temperature coefficient is positive or negative. This negative correlation between the constant and the temperature coefficient suggests that relatively highly productive cows are sensitive to heat.

Many farms use the milk fat ratio as an indicator of milk quality. We rewrite the milk fat ratio, *z*^′^, from Equation (4) as

where *γ*_*i*_=*β*_*i*_−*α*_*i*_, *r*_*i*_=*b*_*i*_−*a*_*i*_, *u*_*j*_(*x*_*jt*_)=*t*_*j*_(*x*_*jt*_)−*s*_*j*_(*x*_*jt*_) and *η*_*it*_=*ξ*_*it*_−*ε*_*it*_. Figure 2 shows the variation of the milk fat ratio according to heat; the intercept *γ*_*i*_ is plotted against the temperature coefficient, *r*_*i*_. The plot also shows a negative correlation, indicating that temperature has a greater effect on the cows that produce milk with a higher milk fat ratio. A negative temperature coefficient indicates a decrease in the milk fat ratio, while a positive one indicates an increase in the milk fat ratio when the maximum temperature increases.

We have found that there are three main scenarios responsible for a decrease in the milk fat ratio: (1) a decrease in milk fat and an increase in milk production (Case 1, *b*_*i*_<0<*a*_*i*_); (2) an increase in milk fat and milk production, but a relatively faster increase in the latter (Case 2, *a*_*i*_>*b*_*i*_>0); and (3) a decrease in milk fat and milk production, but a relatively faster decrease in the former (Case 3, *b*_*i*_<*a*_*i*_<0). The reverse three scenarios are responsible for an increase in the milk fat ratio (Case 4, *a*_*i*_<0<*b*_*i*_; Case 5, *a*_*i*_<*b*_*i*_<0; and Case 6, *b*_*i*_>*a*_*i*_>0).

**Figure 3:**
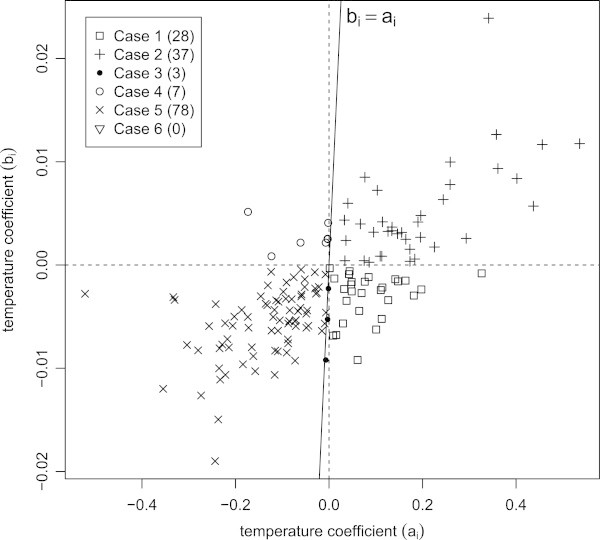
**Scatter plot of the temperature coefficients from milk production (** ***a***_***i***_ **) and milk fat (** ***b***_***i***_ **).** There are six possible scenarios (cases) causing a decrease or an increase in the milk fat ratio. These cases are distinguished by the combination of the signs of the temperature coefficients (*a*_*i*_,*b*_*i*_). Most individual cows fall into one of three distinct cases, Case 1 (28 individuals), Case 2 (37 individuals), and Case 5 (78 individuals).

Figure 3 illustrates how individual cows fall into these six cases, by plotting the temperature coefficient of the milk fat against the temperature coefficient of the milk yield. The solid line (*b*_*i*_=*a*_*i*_) separates the individual cows into two categories according to whether their milk fat ratio increases (left) or decreases (right) as the maximum temperature increases. Clearly, most individual cows fall into one of three cases: Case 1, Case 2, and Case 5. This underscores the fact that for some dairy cows, heat stress leads to an increase in the milk fat ratio. However, few of these cases are caused by an increase in the milk fat yield (Case 4); most are the result of a relatively faster decrease in milk production (Case 5).

### Response curves of milk production and milk fat yield

**Figure 4:**
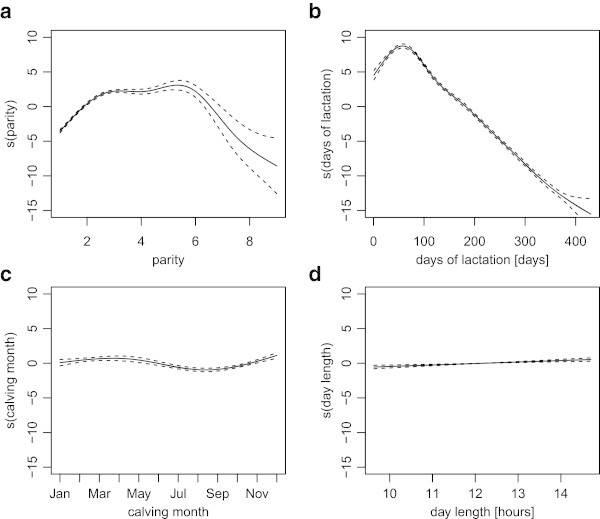
**The estimated response curves of milk production,** ***s***_***j***_ **.** Each panel illustrates how the milk yield responds to different calving or environment factors: **a)** parity; **b)** days of lactation; **c)** calving month; and **d)** day length. The dashed lines are point-wise twice standard-error bands.

Our model also describes how the milk content responds to different calving conditions, such as parity, days of lactation, calving month, and day length. Figure 4 shows the response curve of milk production to each calving condition. The amount of concentrate feed is excluded, as it is dependent upon the amount of milk produced by each cow.

The estimated lactation curve is illustrated in Figure 4b. It shows a typical shape, with a peak around 60 days in lactation followed by a continuous decline. Madalena et al. (1979) report that under intensive production systems in temperate regions such as those existing throughout most of Japan, the lactation curve reaches a peak in week five to six of lactation. In general, however, lactation curves differ according to region. For instance, the lactation curve of European breeds becomes practically linear or has a flat peak (Madalena et al. 1979) in tropical regions; for British herds, the maximum production normally occurs in week five of lactation (Wood 1969).

Milk production also varies according to parity and calving month. Production peaks around the fourth lactation (Figure 4a). In comparison with days of lactation and parity, calving month had a smaller effect on the lactation curve (Figure 4c). As Barash et al. (2001) report, the lowest production occurs in summer, and the highest in winter. We have also investigated the photoperiod effect, that is, varying daylight length. Figure 4d shows a slight increase trend according to longer day length, but its influence is muted in comparison with parity and days of lactation.

**Figure 5:**
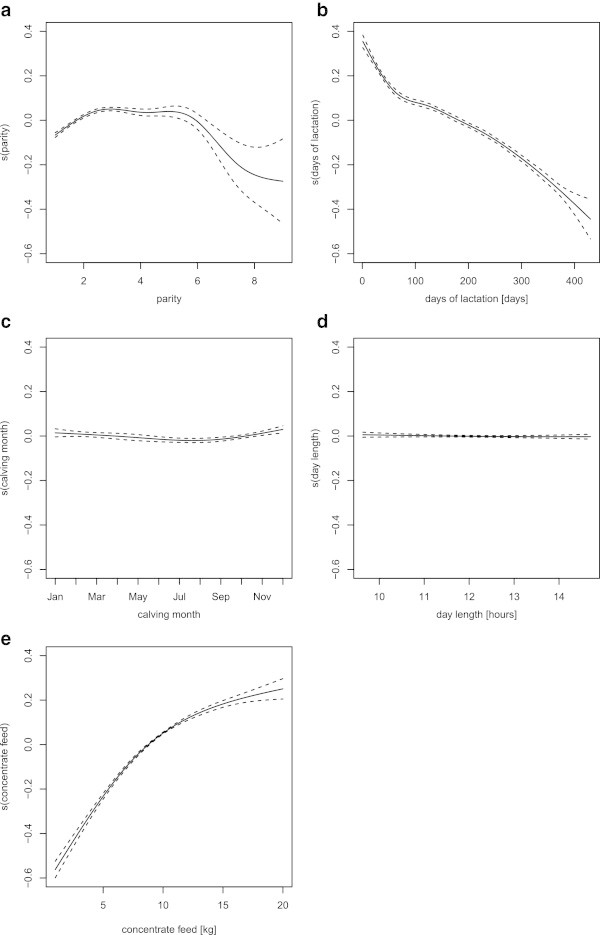
**The estimated response curves of the milk fat yield,** ***t***_***j***_ **, with point-wise twice standard-error bands.** Each panel illustrates how the milk fat yield responds to different calving or environment factors: **a)** parity; **b)** days of lactation; **c)** calving month; **d)** day length; and **e)** the amount of concentrate feed. The dashed lines are point-wise twice standard-error bands.

Figure 5 shows the response of the milk fat component to the conditions of parity, days of lactation, calving month, the amount of concentrate feed, and day length. The responses to parity (Figure 5a) and calving month (Figure 5c) are similar to those shown by milk production; the response curve to parity also shows a peak at the second to fifth lactation. As Barash et al. (2001) report, calving month has a smaller effect on the milk fat component, with the lowest milk fat yield occurring during summer. The response of the milk fat component to days of lactation (Figure 5b) differs most from that of milk production. The fat component is richest at the start of lactation, falls sharply until around 60 days, and thereafter continues to decrease, although relatively more slowly than in the first 60 days. The effect of daylight length (Figure 5d) also shows an almost flat trend. The response curve to concentrate feed increases gradually, indicating that higher feed intake triggers increased production of milk fat.

## Discussion

### Relation to previous studies

#### Lactation curves

Our model is a direct extension of traditional lactation curve models. The early study of the lactation curve can be found in Gaines (1927) and Vujicic and Bacic (1961) and then Wood (1976) refine the traditional lactation curve model. There have been extensive studies undertaken since then (Gnanasakthy and Morant 1990; Goodall 1983; Lannox et al. 1992; Wood 1969; Wood 1972; Wood 1976). Wood (1976) describes milk yield in a non-linear manner:5

where *a*,*b*, and *c* are parameters to be estimated and *η*_*t*_ is an error term. Note that this parametric model is a function of the time *t* since calving.

The non-linear model shown in Equation (5) generally works well, but only takes into account the time since calving. Many model extensions have been proposed that allow the parameters to vary according to different conditions, such as seasonal variation (Gnanasakthy and Morant 1990; Goodall 1983; Wood 1972; Wood 1976) regional variation (Gnanasakthy and Morant 1990), and livestock diet (Lannox et al. 1992).

Our present model provides a more flexible framework, which encompasses the Wood model and its extensions as special cases. For example, taking a natural logarithm of Equation (5), it can be written as

where *ε*_*t*_= log(*η*_*t*_). By comparing this with Equation (2), and rewriting the time since calving as , we obtain

Clearly, our model has extended the lactation curve model, re-parameterising parameters *a*,*b*, and *c* in a more flexible manner. This re-parametrisation provides enhanced modelling flexibility. First, the constant term, log(*a*), is able to cope with the variation originating from factors such as temperature and different calving conditions. Second, the traditional lactation curve is now described as a nonparametric function, , the shape of which can be estimated from the data.

#### Random regression test-day models

Random regression models for test-day data have become increasingly common in animal breeding research. Our model is also related to this modelling approach. A large number of applications can be found in the analysis of the genetic evaluation of dairy cows; see Schaeffer (2004) for a concise review of the model in this area. The basic form of the model consists of three parts: random effects, fixed effects, and an error term, and these terms are accordingly described in the model form as6

for the milk yield *y*_*it*_ of the *i*-th individual at time *t*, for example. On the right-hand side of the model (Equation (6)), the first term, random effects, has a linear form, and each parameter *A*_*ij*_ is assumed to be normally distributed with mean zero (E[*A*_*ij*_]=0) and a constant variance (); the second term, fixed effects *f*_*j*_(*x*_*jt*_) (including a constant term *f*_1_(1)), can have a linear or non-linear form (but is often linear); and the error term *ε*_*it*_ is the Gaussian error with mean zero, but it is not identical; the variance of the error differs in individual cows (), but they are uncorrelated ( for *i*≠*i*^′^). In the context of random regression test-day models, the random effect often represents two effects, genetic and permanent environmental effects. The construction of the model also relies on its variance-covariance structure, for which a variety of structures are available.

Taking *z*_*i*1*t*_=1,*z*_*i*2*t*_=*w*_*t*_ (accordingly *A*_*i*1_=*α*_*i*_ and *A*_*i*2_=*a*_*i*_) and *f*_*j*_=*s*_*j*_, it is clear that the random regression test-day model becomes almost identical to our model (Equation (2)) except for the fact that parameter *A*_*ij*_ is assumed to be normally distributed; our model does not assume any distributions for the parameters, but instead estimates them for each individual cow as  or ). They are fixed effects, in other words. This is the essential difference between the two models. However, it is interesting to note that this makes little difference in the estimation, although it does make a difference in the prediction. For example, the random regression test-day models cannot distinguish individual cows by parameter *A*_*ij*_ as we have done and discussed in the Results section. In contrast, our model cannot give a prediction for absent cows in the data because the individual-dependent parameters are inestimable for unobserved cows. There is no single answer of the question of which model is actually ‘correct’; the choice is largely dependent on the research question. If it aims to predict for a general population of cows regardless of whether they are observed or not, then the random regression test-day model would be more appropriate, but if it intends to distinguish individual cows, as we have discussed, then our model becomes a more suitable candidate.

### Effects of temperature on individual dairy cows

Our present results highlight the importance of investigating individual differences. Although it is beyond our present study, it is likely that such differences, even within the same species, are somehow related to genetic differences. A number of studies on Holsteins have investigated the interaction between genotypes and environmental conditions. Ravagnolo et al. (2000) conclude that considerable genetic variation exists within the Holstein breed. Our model is, however, still able to cope with such genetic differences indirectly as a constant effect, allowing the intercept to differ between individual cows, even though we have no genetic data to characterise individuals. This is the virtue of our modelling approach.

### Management indications

In comparison with milk production, the variation of milk fat content is relatively small. Thus, the milk fat ratio resembles the reciprocal of milk production, as shown in Equation (1). This fact vindicates an empirical finding by Wood (1976). Of course, there is a variety of choices of which indicator to use for management, and it is absolutely the farm’s choice. If the milk fat ratio tends to be preferable, the six different scenarios leading to a variation of the milk fat ratio provide useful indications for management planning strategies. Although management actions to reduce the negative effects of heat cannot be applied to each individual within a large production system (André et al. 2011), our present analysis highlights only six different required treatment strategies. Further, these may be reduced to the three major cases shown in Figure 3. Appropriate management action can be taken regarding feed composition and the prioritised allocation of cows in the barn. For Case 2, in which the production of milk and milk fat increase, no special treatment is actually required despite a decrease in the milk fat ratio. The reason for this is the faster increase of milk production compared to that of milk fat yield. For Case 5, cows are strongly affected by heat, but the milk fat ratio increases. The decreased production of milk and milk fat may be offset by allocating the cows as cool a space as possible and providing them with easily digestible and high-calorie feed. For Case 1, the decreased production of milk fat may be offset by providing cows with a fat-productive feed.

## Concluding remarks

We have presented a modelling framework for milk production and its fat component from individual dairy cows by extending both the traditional lactation curve model (Wood 1976) and random regression test-day data models (Schaeffer 2004) onto a more flexible statistical modelling framework, GAM. The GAM allows simultaneous modelling of various calving conditions in an appropriate non-linear structure. Our model has shown clear evidence that cows producing high quantities of milk are sensitive to heat and tend to decrease their milk production as the temperature increases. However, some individuals relatively increase their milk production as the temperature increases.

Our analysis has suggested that the milk fat ratio is dependent upon and driven by the variation of milk and milk fat production according to heat. We have identified six distinct scenarios that underpin an increase or a decrease in the milk fat ratio. Our results indicate that efficient managing strategies are required for each group; varying the feed composition may be effective.

Given the retrospective nature of our study data, we are unable to determine whether the variation in milk production is directly driven by high temperature itself or whether a high temperature indirectly triggers poor feed supply. Nevertheless, by revealing different scenarios leading to a variation in the milk fat ratio, our model provides useful indications for management planning strategies. The model can also be applied to milk components such as the protein yield and protein ratio (also a common indicator of milk quality). Moreover, providing that sufficient data are available, the model can be used to predict future milk production and composition.

## Materials and methods

### Data

Throughout this paper, we focus on two data sets: (i) the test-day data and (ii) the environment data, which include daily maximum temperature records and daylight length for the studying period (1989–1998) at Jiyu Gakuen Nasu Farm (36° 56^′^N, 139° 58^′^E) in Tochigi Prefecture, which has the second-largest dairy cow population in Japan.

The test-day data for individual dairy cows comprise six items, namely, milk yield, milk fat ratio (the amount of milk fat is not given), parity, days of lactation, calving month, and amount of concentrate feed (see Table 1 for the summary statistics). The test is undertaken and reported every month by the Livestock Improvement Association of Japan Inc.

**Table 1:** **Summary statistics of the test-day data**

	Min	Median	Mean	Max
Milk production [kg]	3.20	26.20	26.33	53.00
Milk fat ratio [%]	2.20	3.80	—	7.90
Parity	1.00	2.00	2.58	9.00
Calving month	1.00	8.00	7.03	12.00
Days of lactation	1.00	173.00	176.10	430.00
Amount of concentrate feed [kg]	1.00	10.00	9.52	20.00

The arithmetic mean is shown for all data except for the milk fat ratio, which is given as a percentage.

We have selected 153 lactating Holstein cows from the farm for which test-day data are available over a minimum of twelve months. The number of data points vary according to individual cows; they comprise between 12 and 65 observations in total for each. Those data all are used to estimate the parameters of the models. The cows are housed in a covered tie stall barn with no cooling system for 20 hours per day. Except when raining, the cows are generally kept outside from 10 a.m. to 2 p.m. All of the cows are milked and fed twice daily, at 5 a.m. and 4:30 p.m. Although the amount and composition of feeds vary depending on cows’ condition, a combination of forages and concentrate feed consisting of carbohydrate and protein (maize and oats (32%), wheat and rice bran, and soy (25%), oil cake of soy and coleseed (10%), and others (33%)) is supplied.

**Figure 6:**
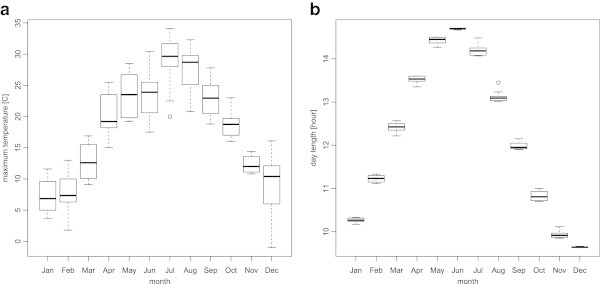
**Monthly variation of the environment factors for the study period 1989–1998.** **a)** the maximum temperature of the test day; **b)** the daylight length (hour) of the test day.

To investigate the effect of temperature, we use the daily maximum temperature recorded on the day of testing by using a maximum-minimum thermometer in an instrument shelter located 20 metres from the dairy barn. The monthly variation shows a typical unimodal trend, with a peak of around 30°C during summer and a trough of around 7°C during winter (Figure 6a). The greatest difference, around 23 degrees, occurs between January and July.

The daylight length of each test day is calculated as follows. Given a solar location on the celestial sphere; that is, the declination and right ascension (*δ*(*d*),*α*(*d*)) of a particular date and time, sunrise and sunset times, *d*, at a geological location (*λ*,*ψ*) on the Earth satisfy the following equation (Nagasawa 1999):

where *t*(*d*)=*Θ*_0_(*d*)+*λ*−*α*(*d*) is the solar hour angle and *k*(*d*) is the solar elevation. The monthly variation of the daylight length of test days is illustrated in Figure 6b; it varies within a five-hour difference (between about 9.5 to 14.5 hours) over a year, which is a narrower variation in comparison with other higher-latitude countries. The monthly variations also show a typical unimodal trend, with a peak at June and a trough at December, the summer and winter solstices. Note that this peak and trough do not coincide with those of the maximum temperature (Figure 6a).

### Parameter estimation

For ease of exposition, we describe the parameter estimation procedure, taking the model of milk yield (Equation (2)) as an example. We rewrite the model using vector notation as

where , for example. The parameters to be estimated here are ***α***,***a***, and the smooth function ***s***_*j*_. In particular, each ***s***_*j*_ is modelled by a smooth spline function (Hastie and Tibshirani 1990). We also assume the heteroscedasticity of ***ε***, which means that the error *ε*_*it*_ is not identical but uncorrelated between individual cows; the covariance matrix of the error, **Ω**, is then given as7

where ***I***_*i*_ is an identity matrix whose diagonal elements are all 1. Note that the variances , (*i*=1,2,…,153) are now also parameters to be estimated. As to the covariance matrix structure here (Equation (7)), it specifically assumes statistical independence within cows over time; no temporal correlations, in other words, are assumed which can be relaxed for future model extension.

To estimate those parameters and smooth functions, we minimise the weighted least squared8

under the GAM framework, recalling that . Here, the diagonal elements of the inverse matrix are reciprocal of each variance, . However, to estimate the variance components, we have to explicitly model the variance heterogeneity. It is known that the normalised squared residual follows the chi-squared distribution with 1 degree of freedom, . As the the chi-squared random variable is twice a gamma variable with 1/2 degree of freedom, we fit a simple generalised linear model (GLM; McCullagh and Nelder 1989) with the gamma distribution, *Γ*(1/2,2), as9

The estimate of variance is then given as .

The estimation algorithm employed is summarised as follows.

1. Apply Equation (8) with ;
2. Repeat steps 3 to 4 until the estimates stop changing;
3. Estimate *τ*_*i*_ by Equation (9);
4. Apply Equation (8) with weight .

We have conducted the analysis and modelling tasks by a statistical computing language R (R Core Team 2013).

### Model diagnostics

Our models (2) and (4) assume a linear relationship between the maximum temperature and each of the milk production and the milk fat as a simple approximation. We inspect whether the assumption made is reasonably appropriate by plotting partial residuals which are defined as

Additional files 1 and 2 respectively show the partial residual plots of the milk production, , and of the milk fat, , for each cow. We have interpreted these plots as that the majority of the cows, although there are of course some exceptions, appear to have a linear relationship with the maximum temperature rather than non-linear of a particular form.

To assess the goodness of fit of our models, we plot the fitted values of the milk production (Additional file 3) and of the milk fat (Additional file 4) in the natural log scale for each cow, along with the observations. The superposed red line in each panel represents the fitted values. Based on this visual assessment, we regard that while our model is not perfect, it reasonably represents the data observed. Although there are some observations lying slightly away from the fitted value, we for now leave them for further investigations in the future.

## Electronic supplementary material

Additional file 1: **The partial residual plots of the milk production,**  **, for each cow.** (PDF 77 KB)

Additional file 2: **The partial residual plots of the milk fat,**  **, for each cow.** (PDF 76 KB)

Additional file 3: **The fitted values of the milk production in the natural log scale for each cow, along with the observations.** The superposed red line in each panel represents the fitted values. (PDF 123 KB)

Additional file 4: **The fitted values of the milk fat in the natural log scale for each cow, along with the observations.** The superposed red line in each panel represents the fitted values. (PDF 124 KB)
