# Supplementary material for: Modelling temperature effects on milk production: a study on Holstein cows at a Japanese farm
Source: Springerplus. 2014 Mar 7;3:129. doi: 10.1186/2193-1801-3-129 (PMC3979979; doi:10.1186/2193-1801-3-129)
Supplement: Supplementary file 1 — Additional file 1: The partial residual plots of the milk production, , for each cow. (PDF 77 KB) [file 40064_2013_878_MOESM1_ESM.pdf]

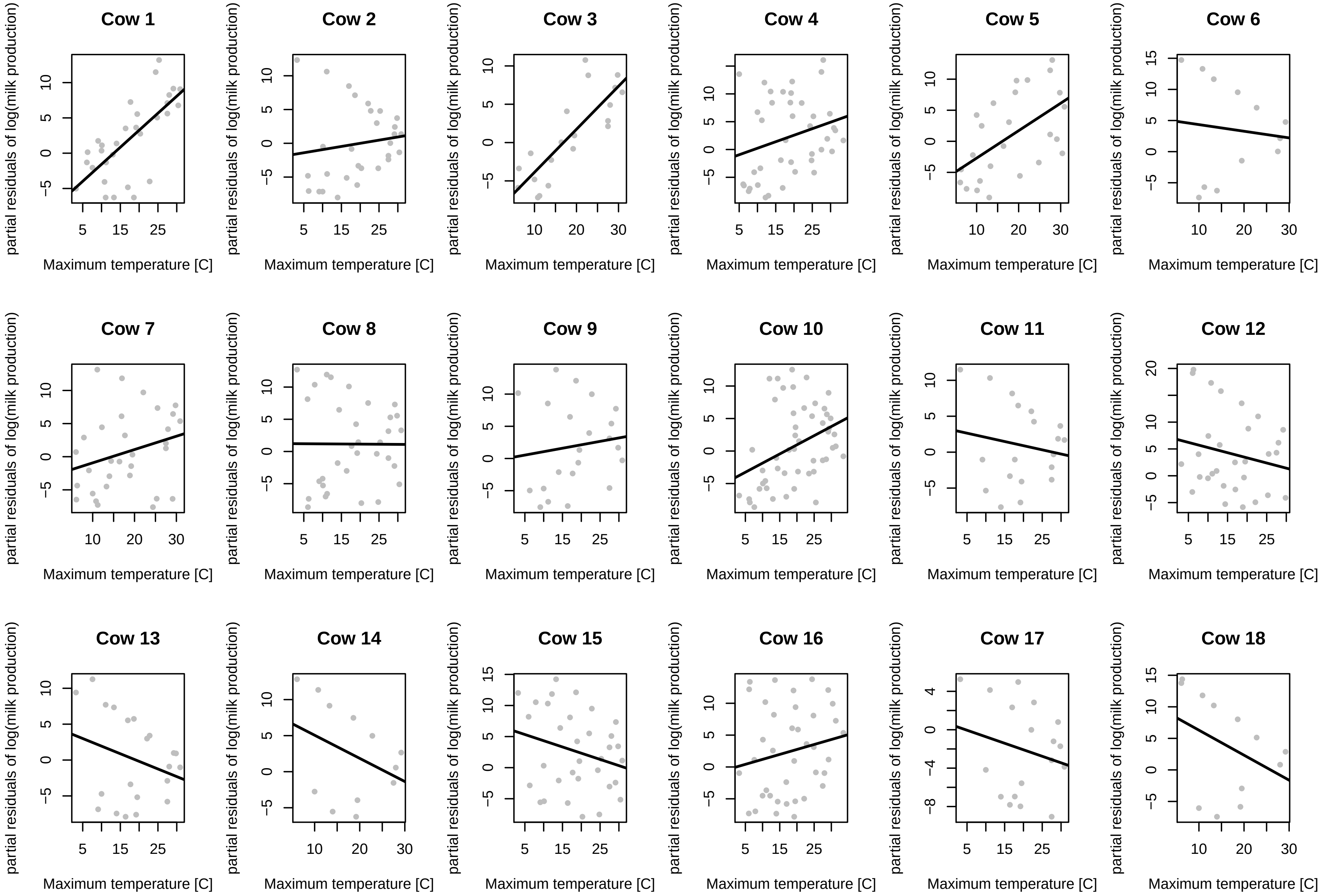
Cow 1
Cow 2
Cow 3
Cow 4
Cow 5
Cow 6
Cow 7
Cow 8
Cow 9
Cow 10
Cow 11
Cow 12
Cow 13
Cow 14
Cow 15
Cow 16
Cow 17
Cow 18
partial residuals of log(milk production)
Maximum temperature [C]

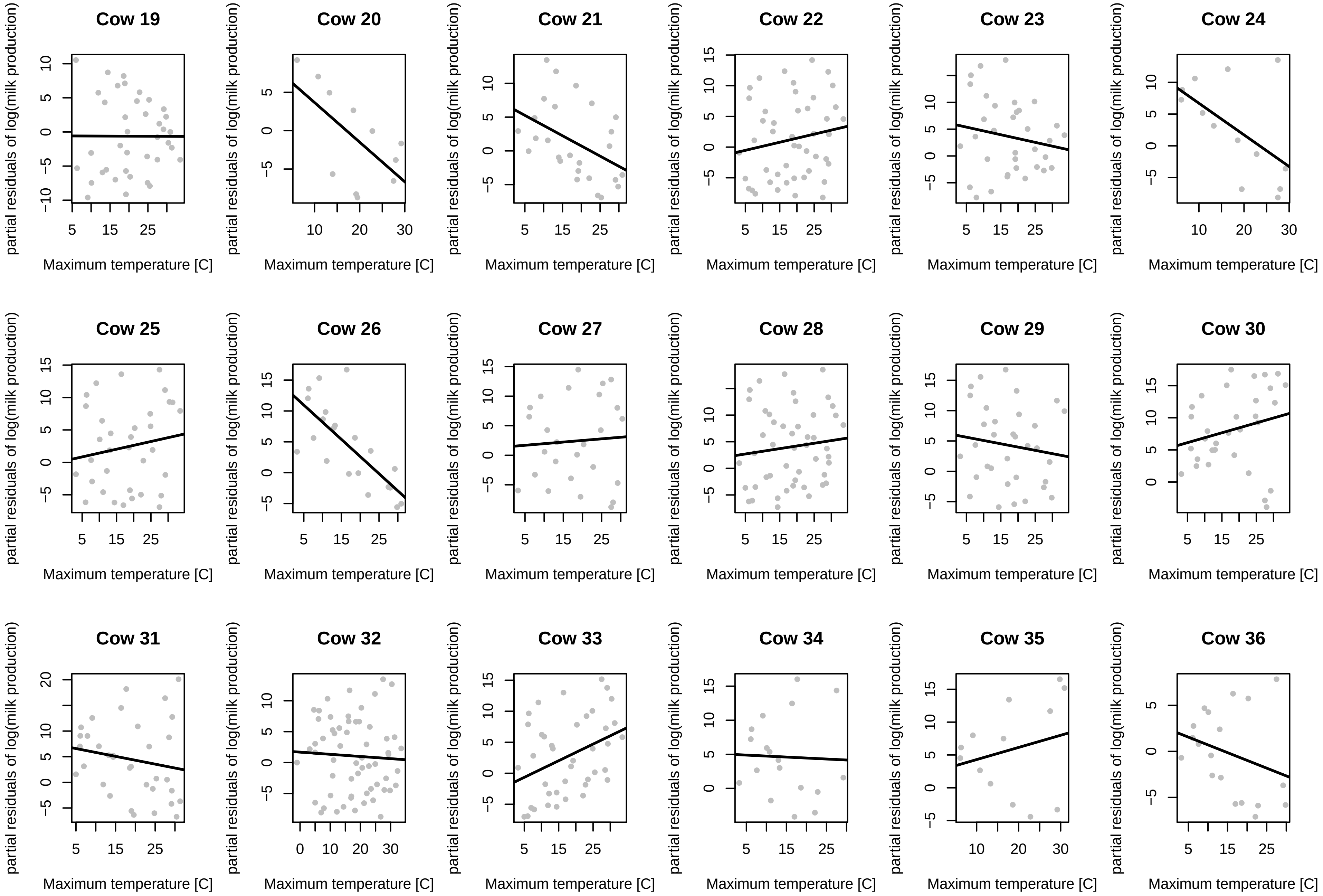
Cow 19
Cow 20
Cow 21
Cow 22
Cow 23
Cow 24
Cow 25
Cow 26
Cow 27
Cow 28
Cow 29
Cow 30
Cow 31
Cow 32
Cow 33
Cow 34
Cow 35
Cow 36
partial residuals of log(milk production)
Maximum temperature [C]

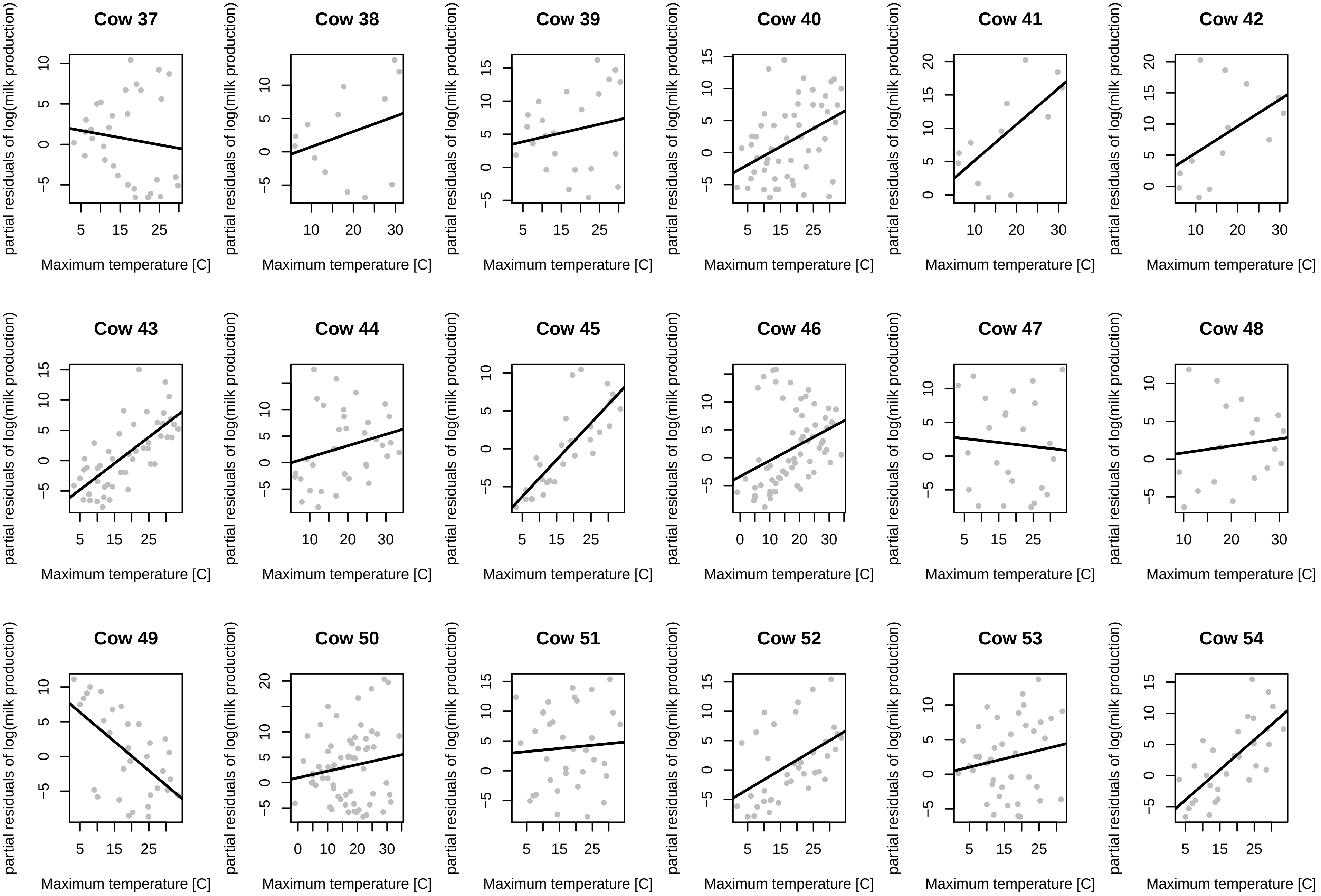

Cow 37
Cow 38
Cow 39
Cow 40
Cow 41
Cow 42
Cow 43
Cow 44
Cow 45
Cow 46
Cow 47
Cow 48
Cow 49
Cow 50
Cow 51
Cow 52
Cow 53
Cow 54
partial residuals of log(milk production)
Maximum temperature [C]

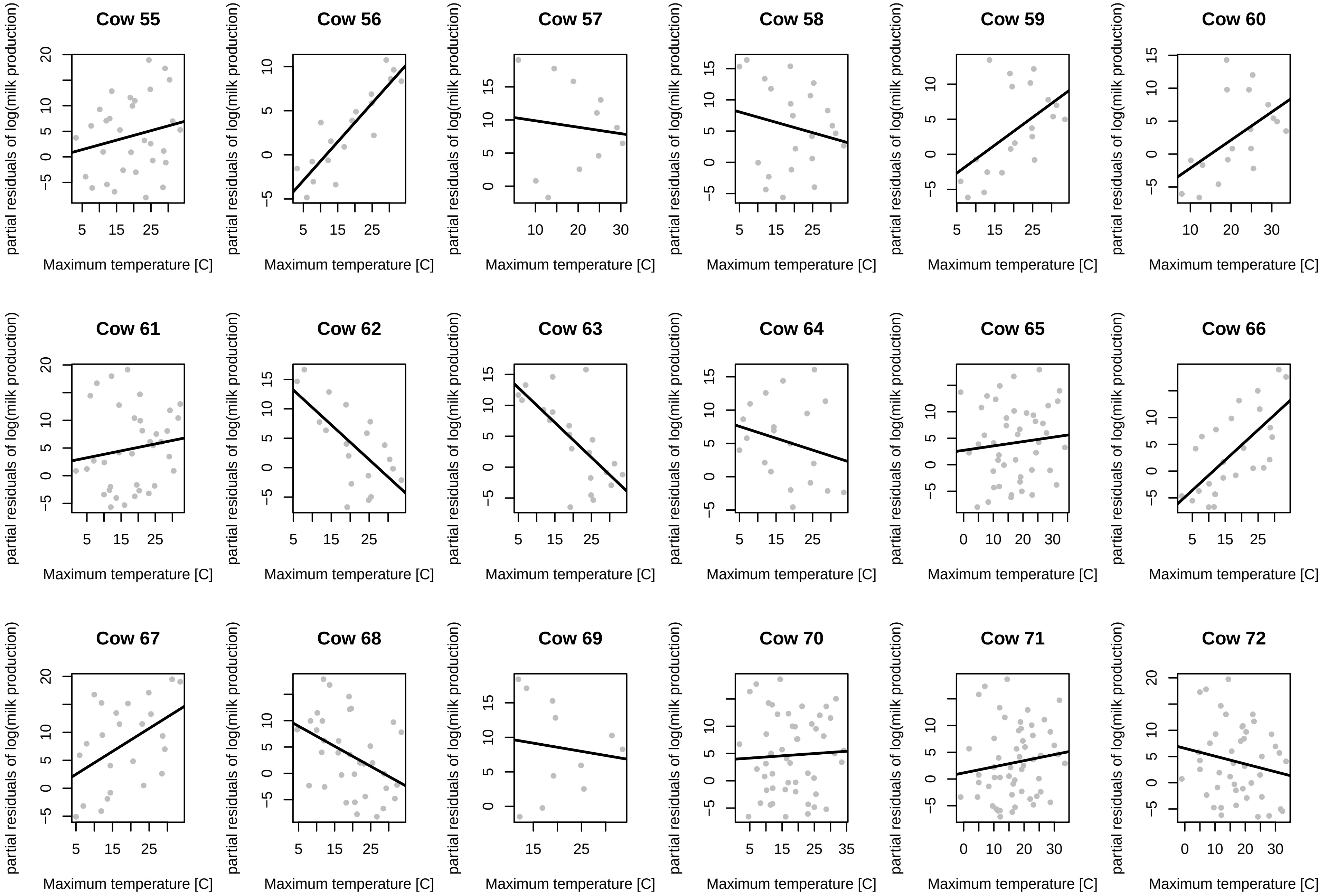
Cow 55
Cow 56
Cow 57
Cow 58
Cow 59
Cow 60
Cow 61
Cow 62
Cow 63
Cow 64
Cow 65
Cow 66
Cow 67
Cow 68
Cow 69
Cow 70
Cow 71
Cow 72
partial residuals of log(milk production)
Maximum temperature [C]

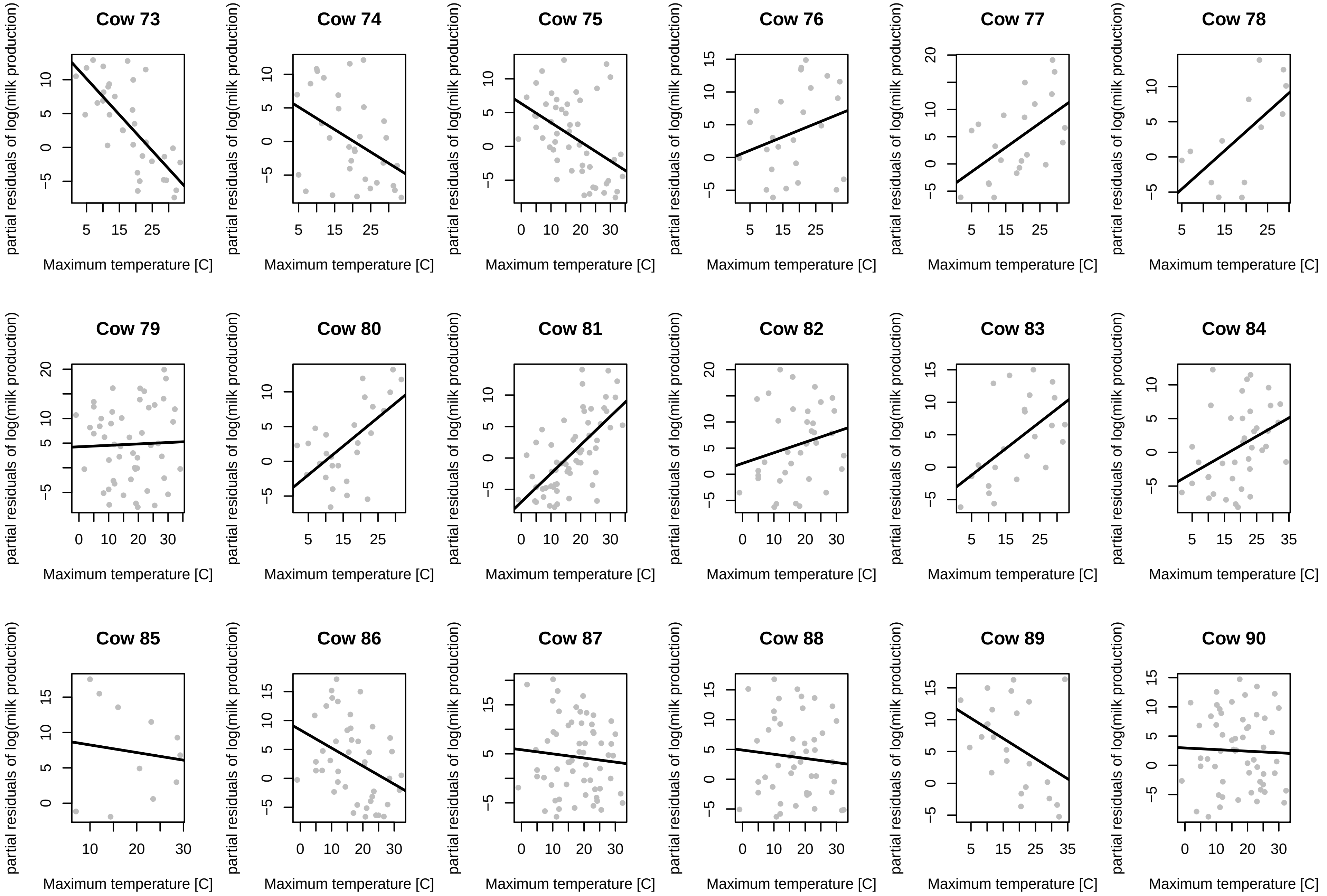
Cow 73
Cow 74
Cow 75
Cow 76
Cow 77
Cow 78
Cow 79
Cow 80
Cow 81
Cow 82
Cow 83
Cow 84
Cow 85
Cow 86
Cow 87
Cow 88
Cow 89
Cow 90
partial residuals of log(milk production)
Maximum temperature [C]

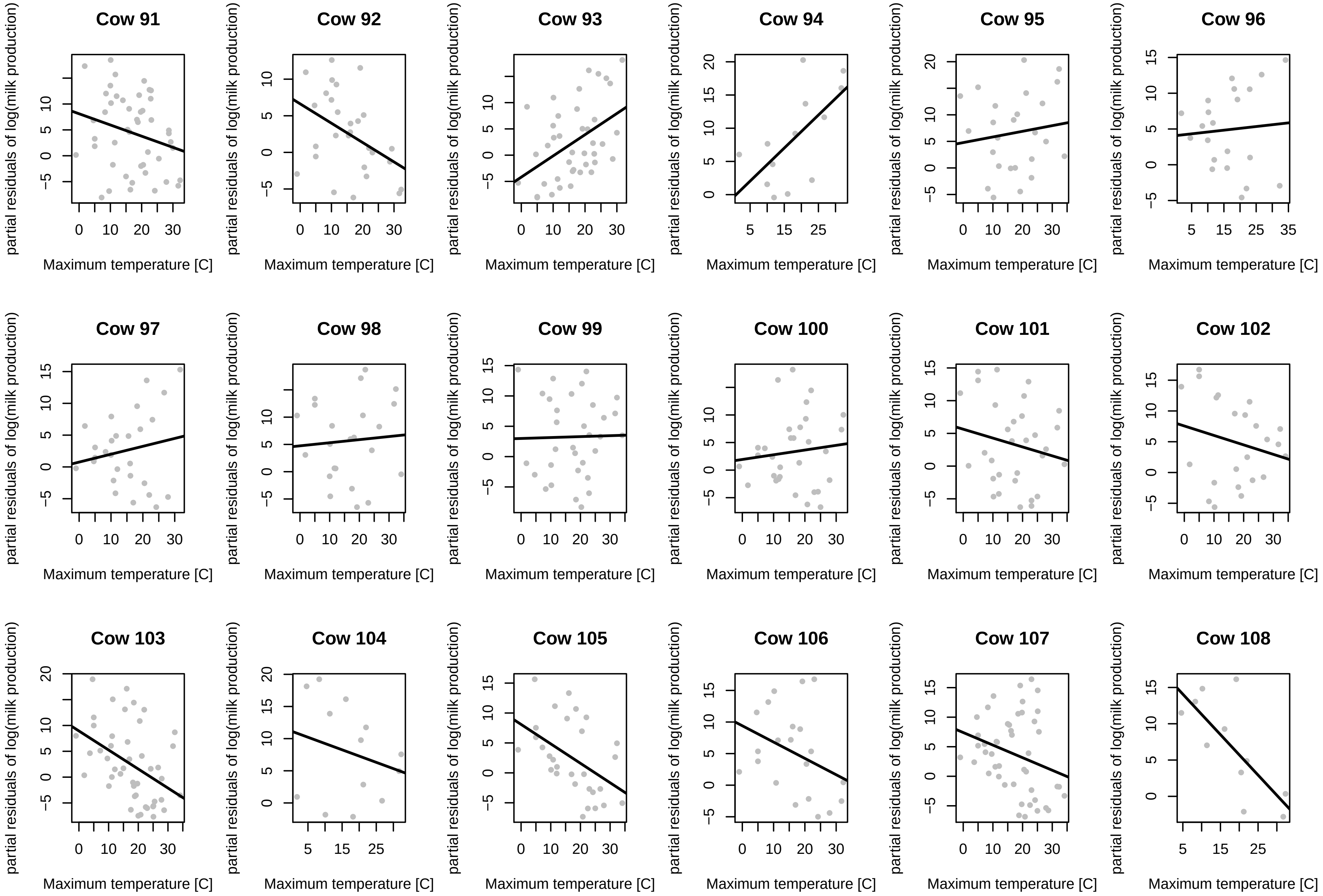
Cow 91
Cow 92
Cow 93
Cow 94
Cow 95
Cow 96
Cow 97
Cow 98
Cow 99
Cow 100
Cow 101
Cow 102
Cow 103
Cow 104
Cow 105
Cow 106
Cow 107
Cow 108
partial residuals of log(milk production)
Maximum temperature [C]

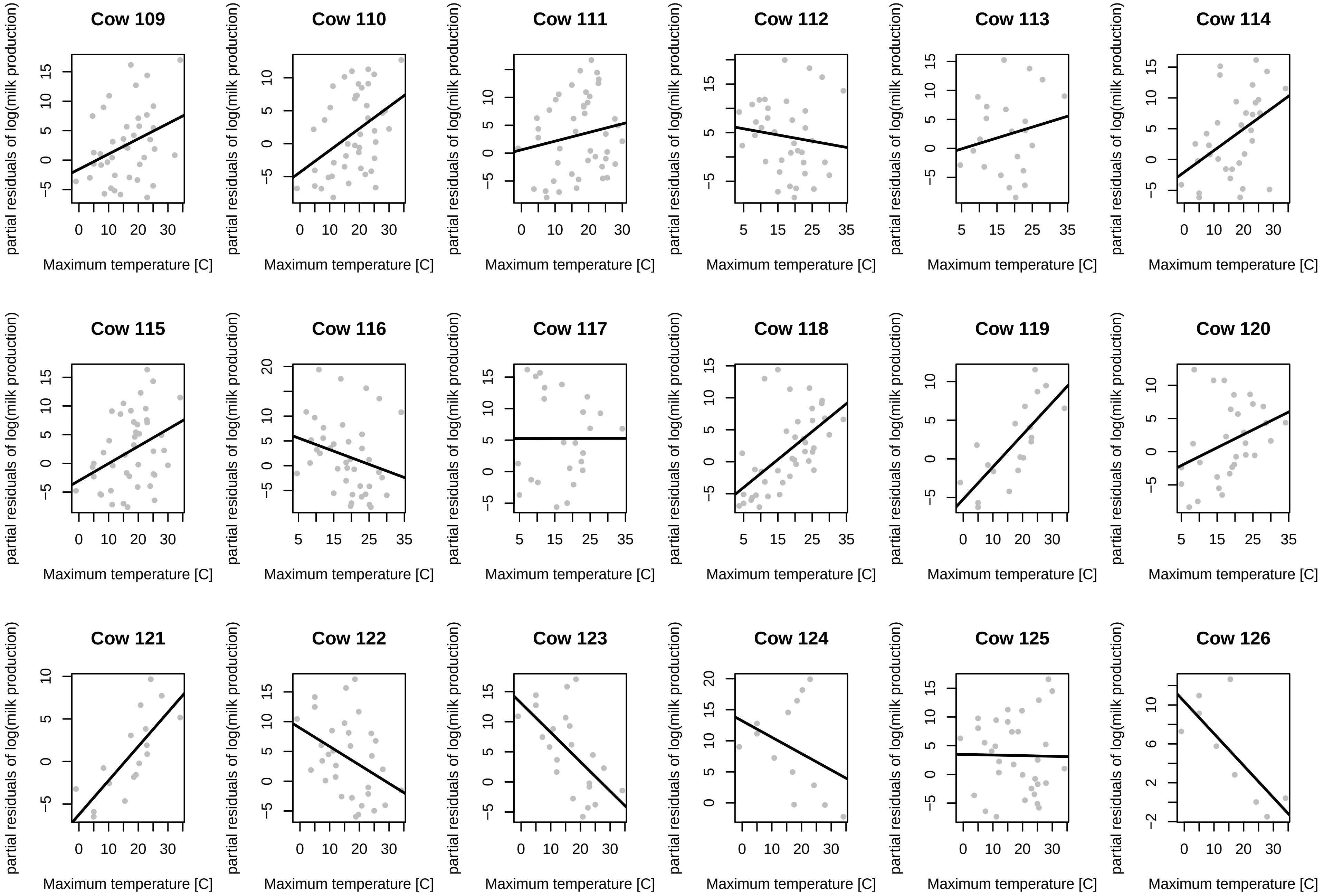
Cow 109
Cow 110
Cow 111
Cow 112
Cow 113
Cow 114
Cow 115
Cow 116
Cow 117
Cow 118
Cow 119
Cow 120
Cow 121
Cow 122
Cow 123
Cow 124
Cow 125
Cow 126
partial residuals of log(milk production)
Maximum temperature [C]

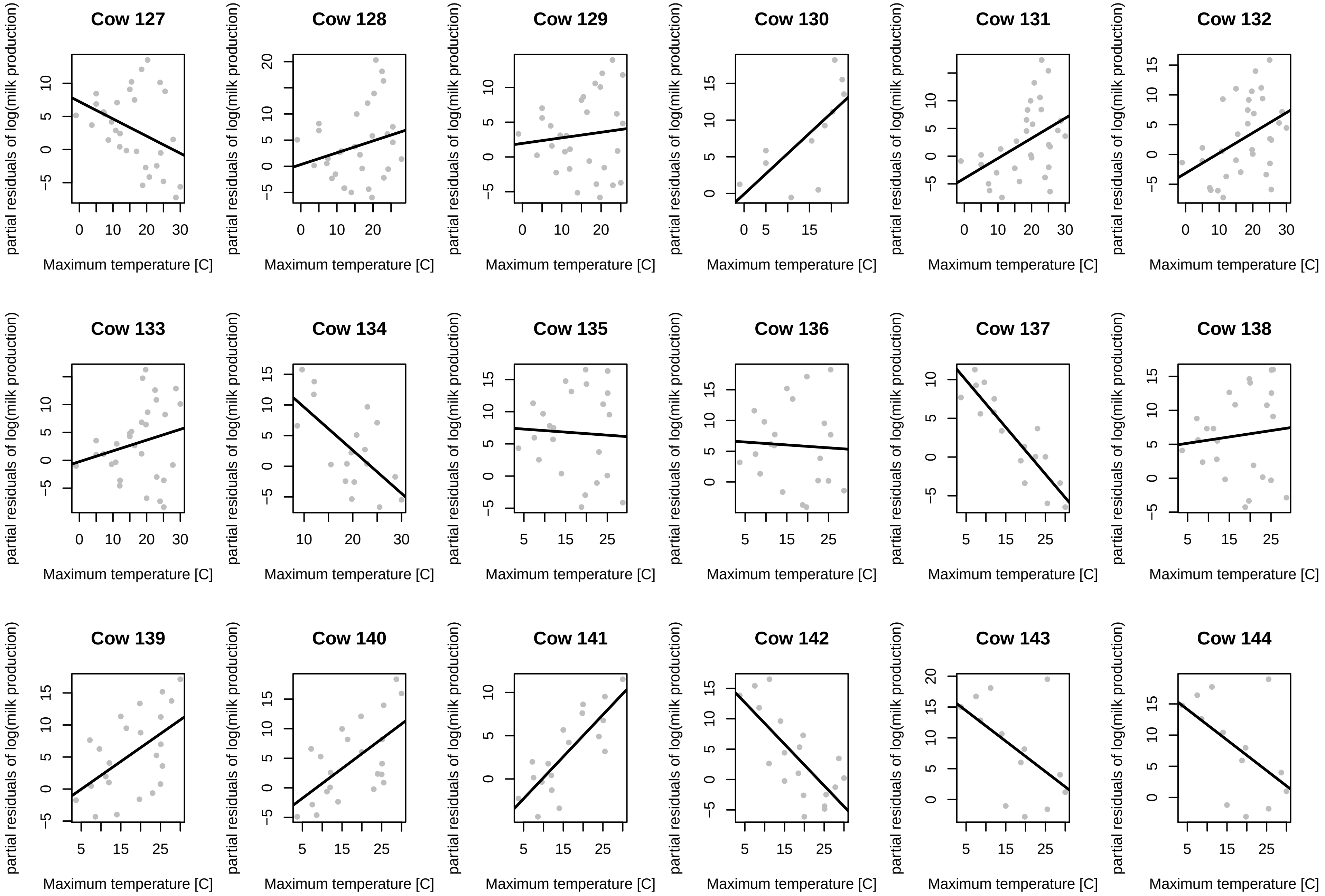
Cow 127
Cow 128
Cow 129
Cow 130
Cow 131
Cow 132
Cow 133
Cow 134
Cow 135
Cow 136
Cow 137
Cow 138
Cow 139
Cow 140
Cow 141
Cow 142
Cow 143
Cow 144
partial residuals of log(milk production)
Maximum temperature [C]

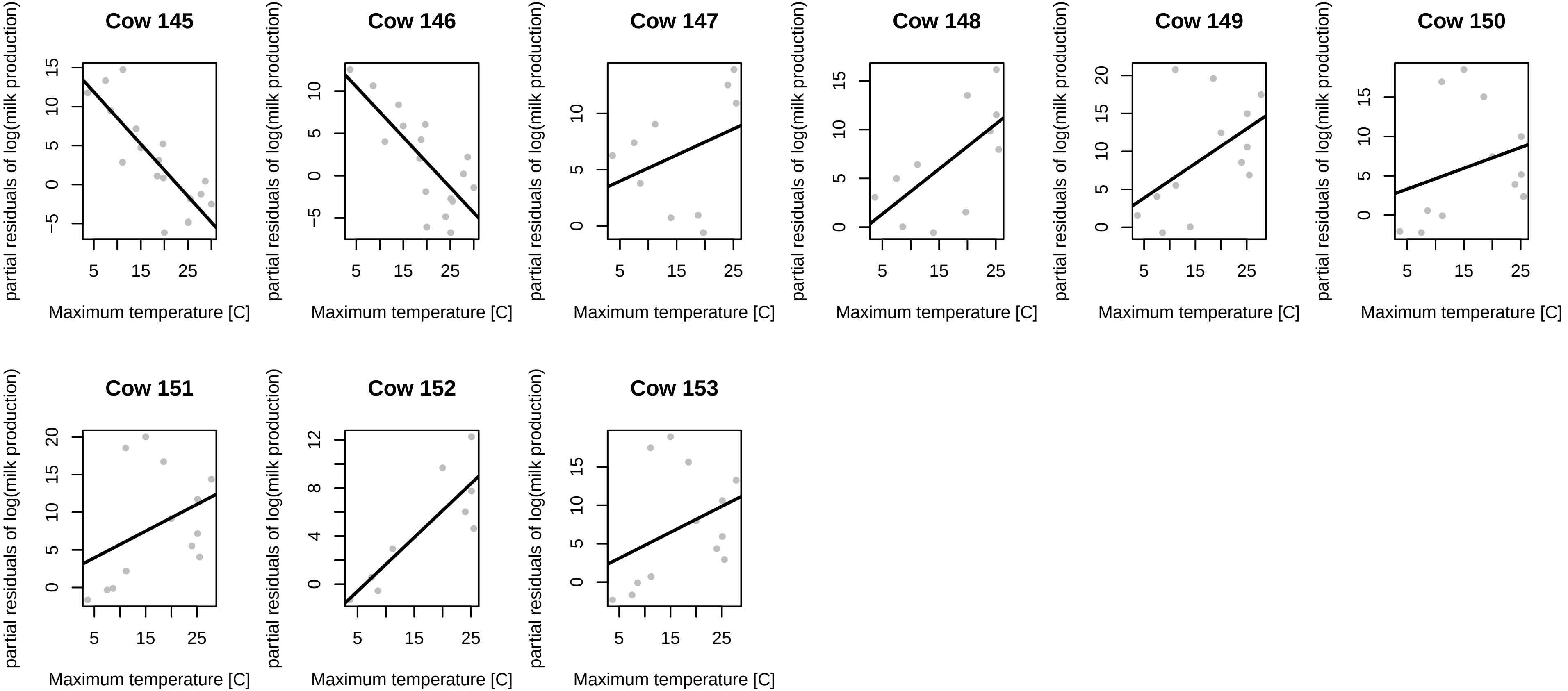
Cow 145
Cow 146
Cow 147
Cow 148
Cow 149
Cow 150
Cow 151
Cow 152
Cow 153
partial residuals of log(milk production)
Maximum temperature [C]
