# Supplementary figures and images for: Modelling temperature effects on milk production: a study on Holstein cows at a Japanese farm
Source: Springerplus. 2014 Mar 7;3:129. doi: 10.1186/2193-1801-3-129 (PMC3979979; doi:10.1186/2193-1801-3-129)

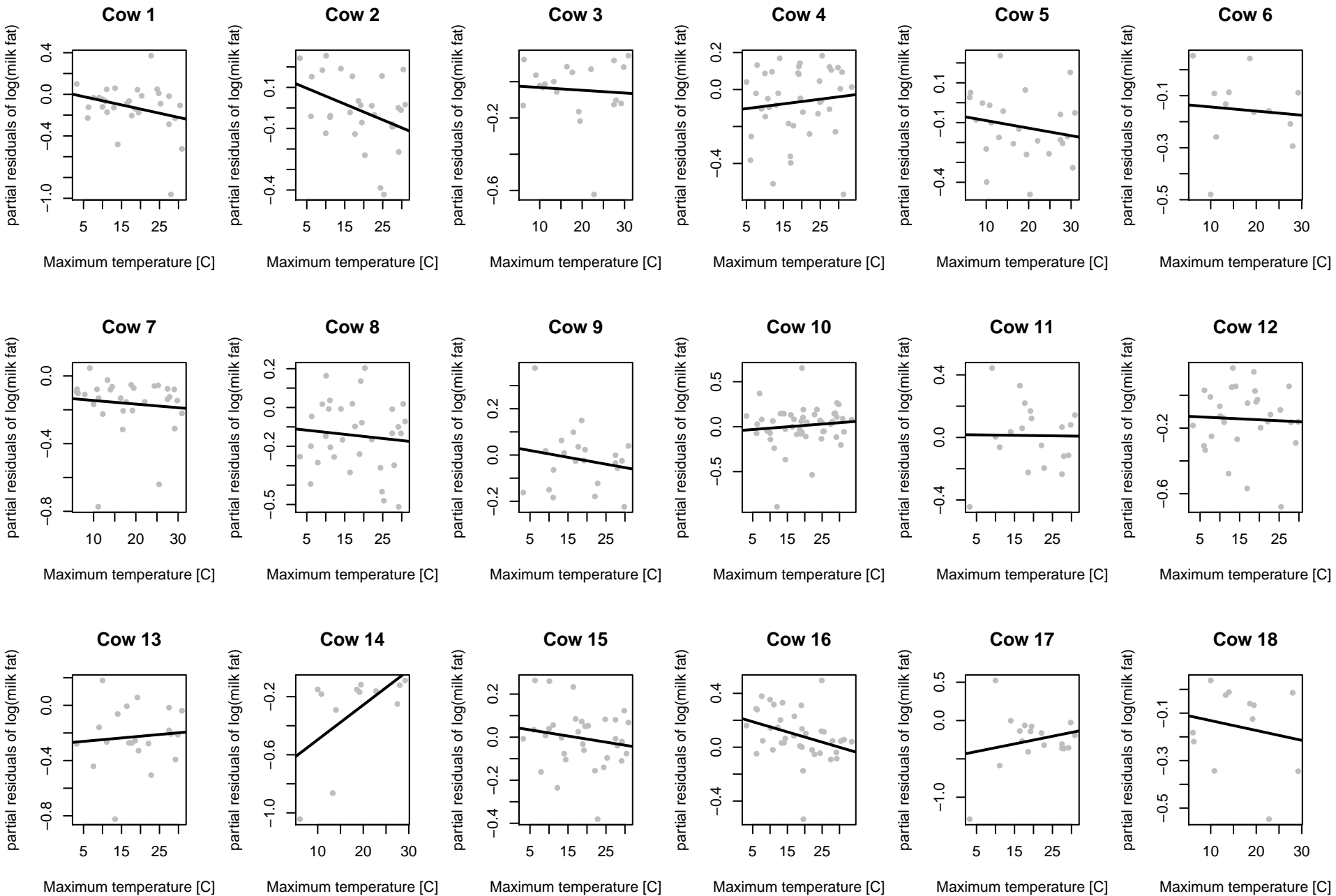

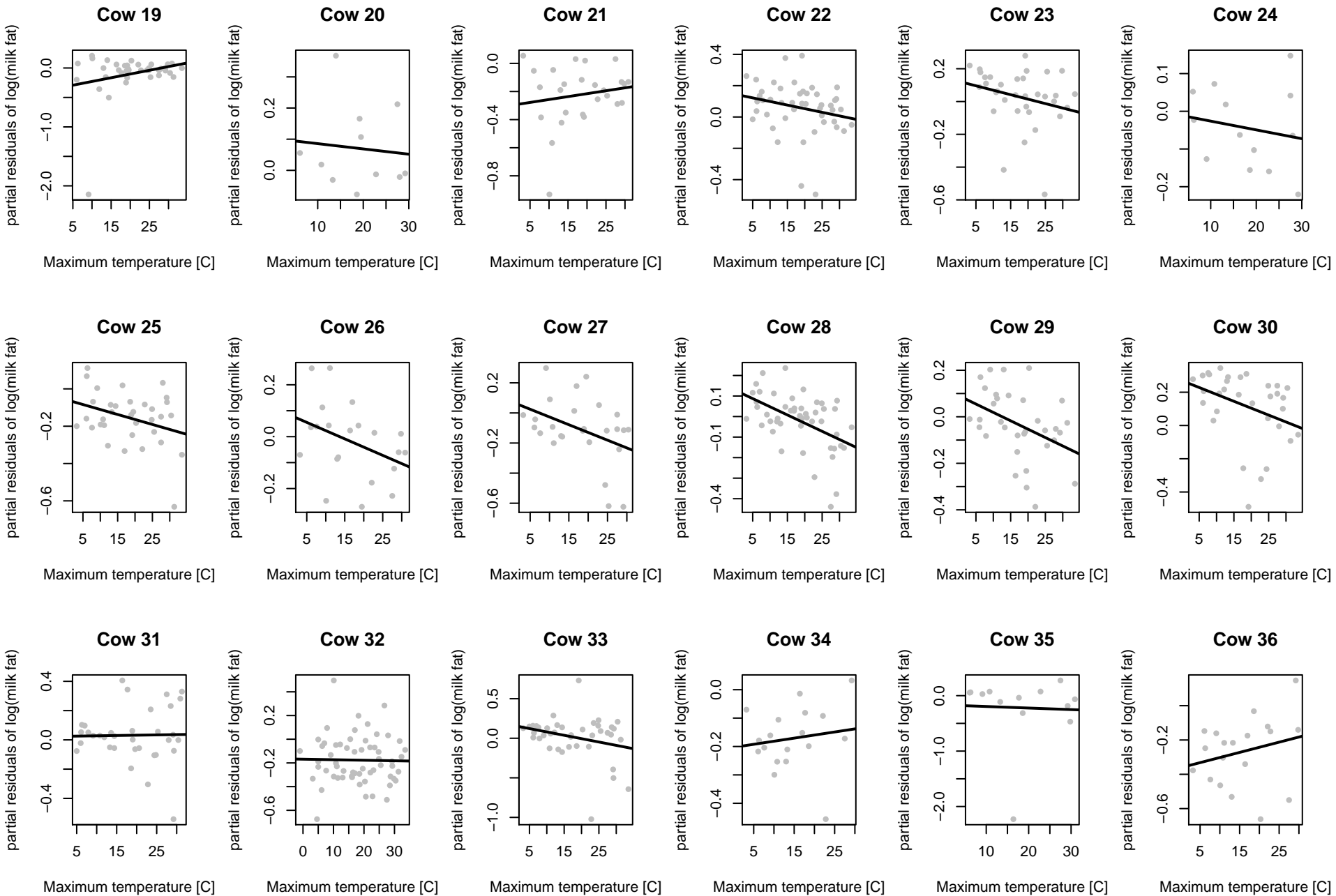

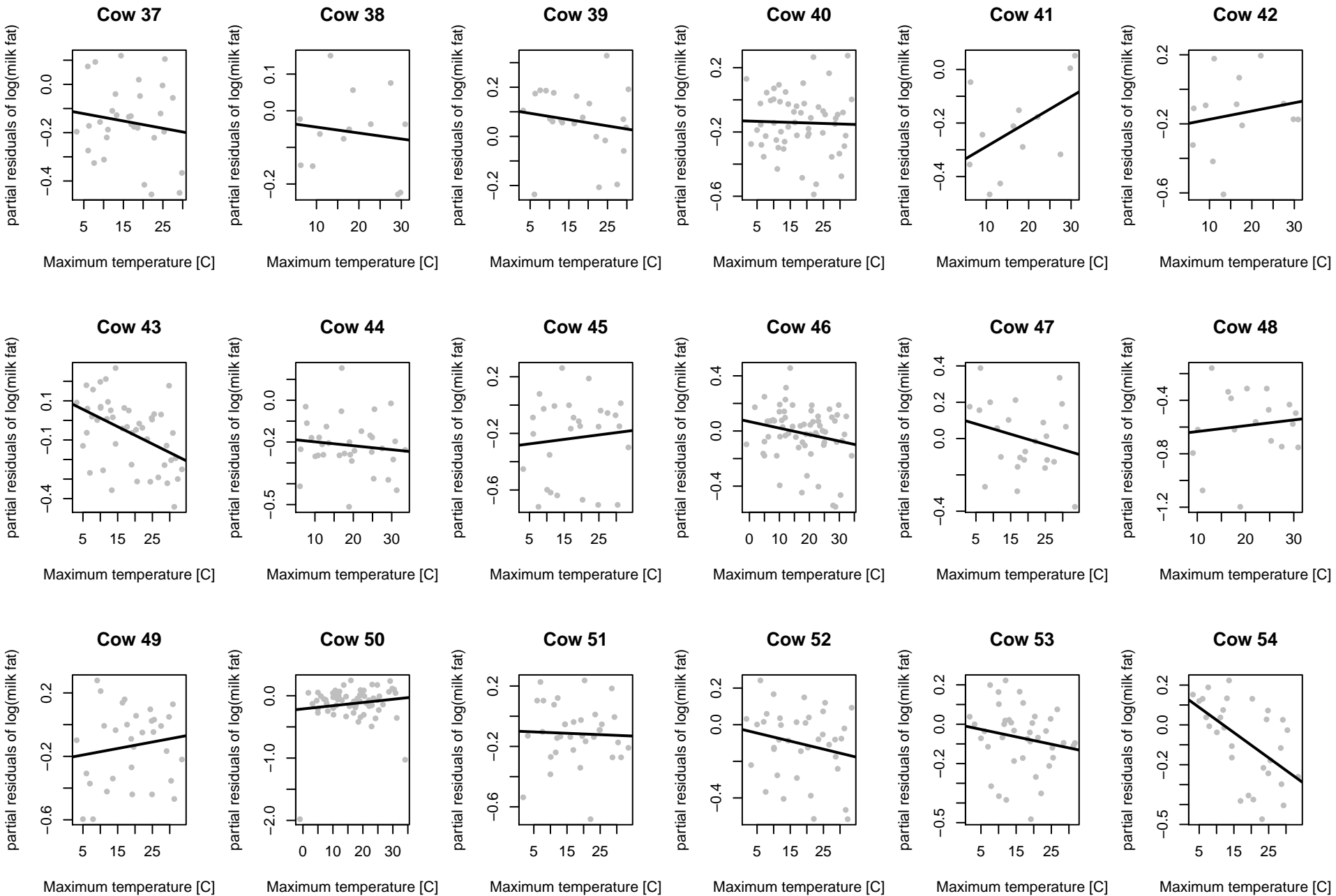

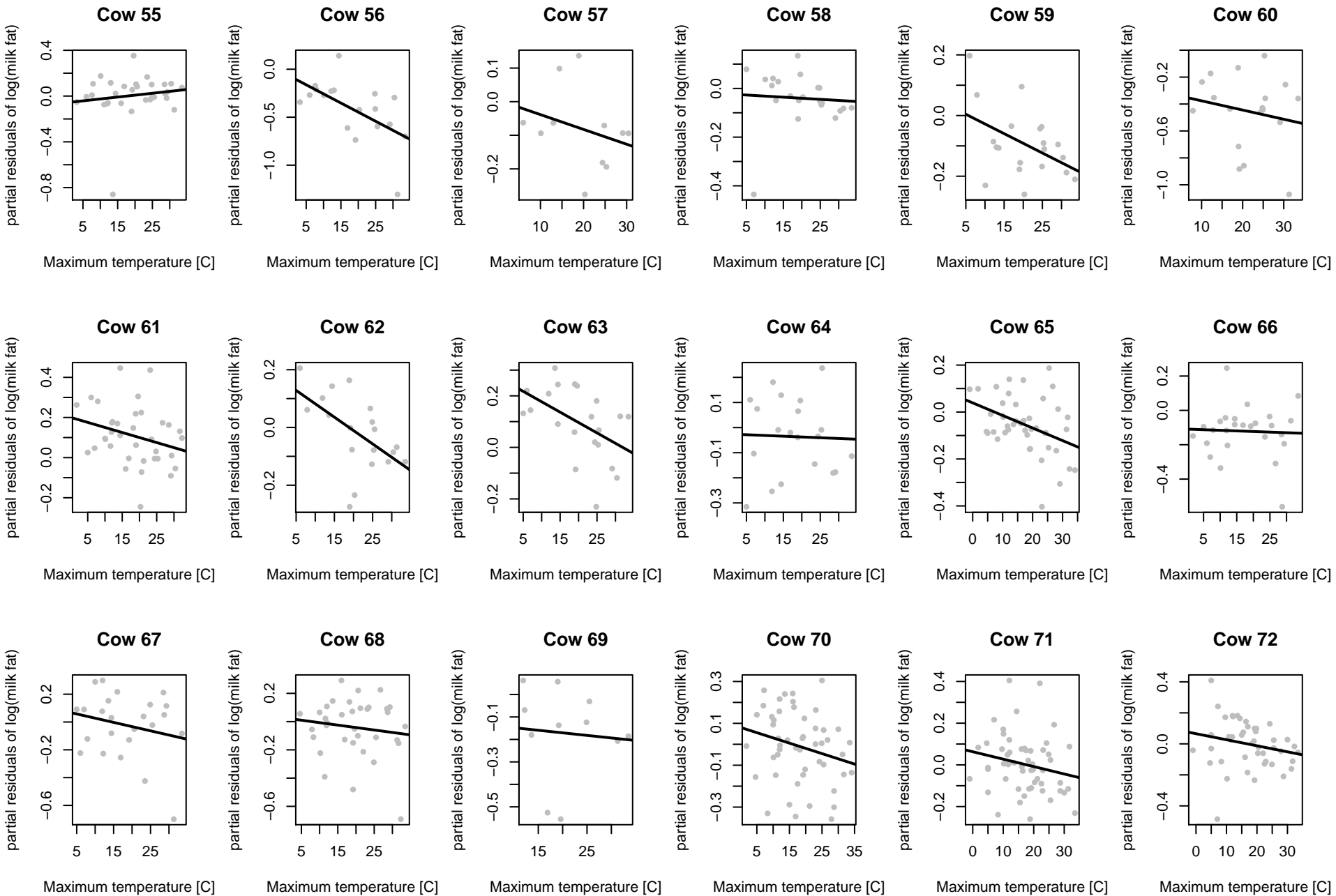

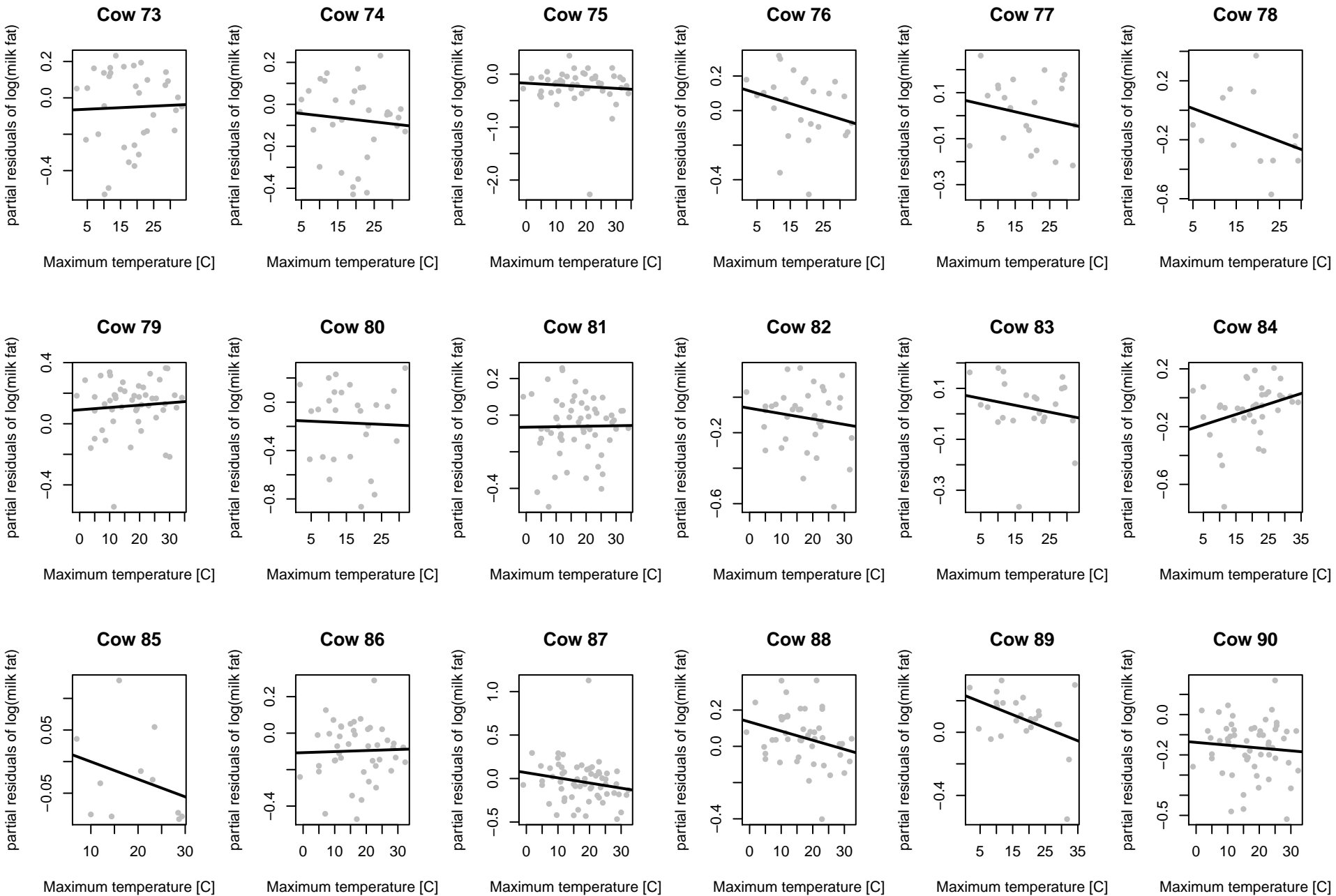

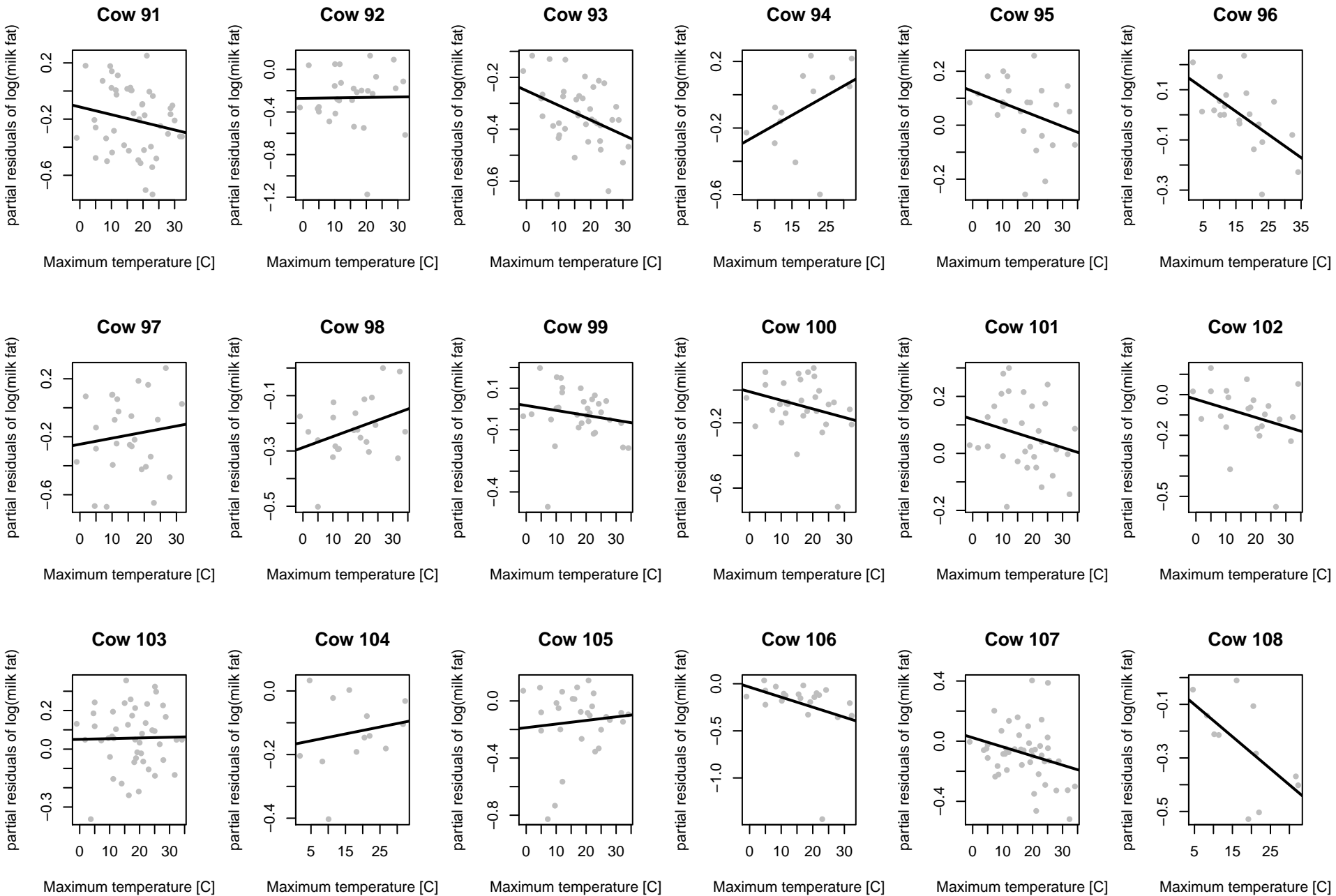

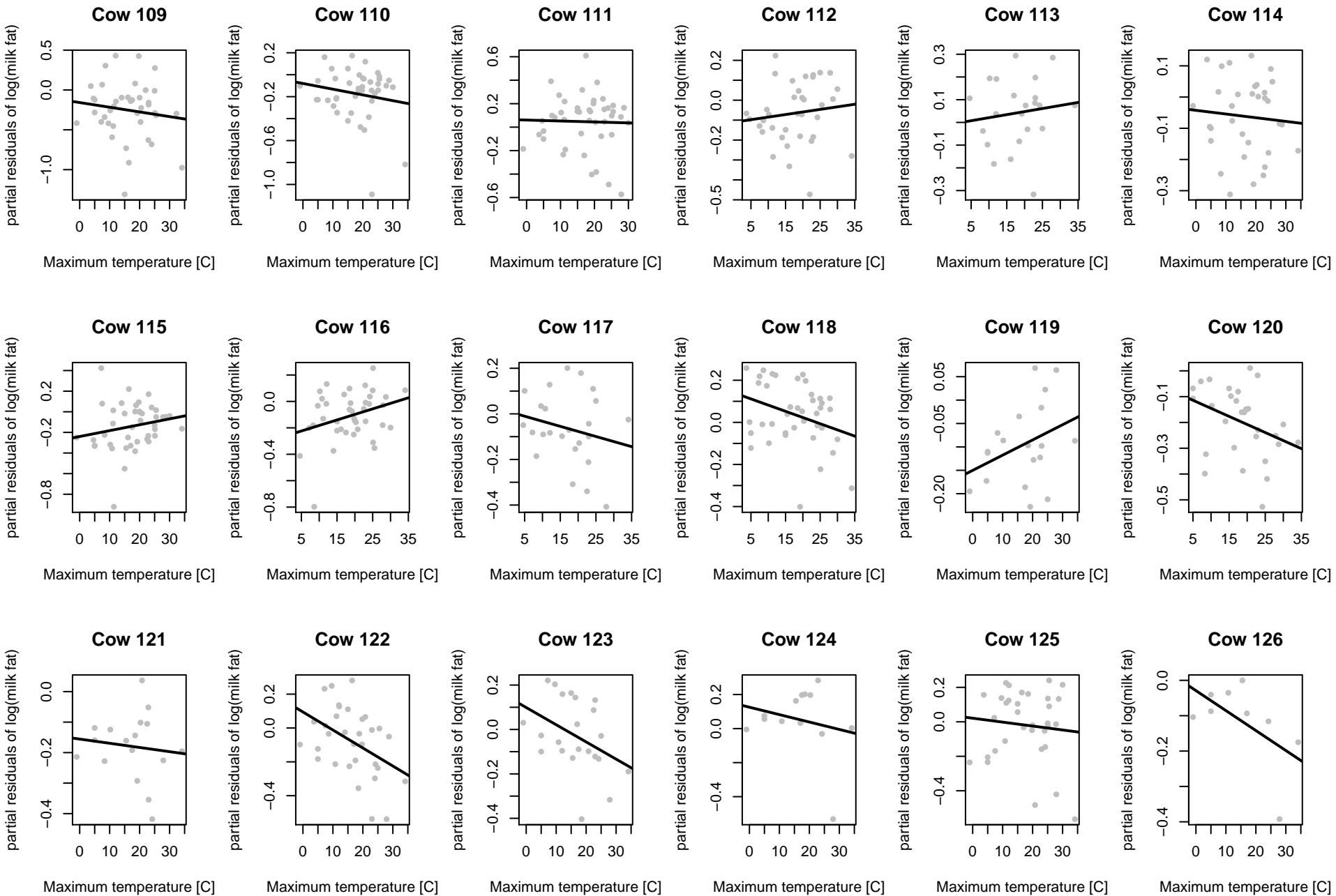

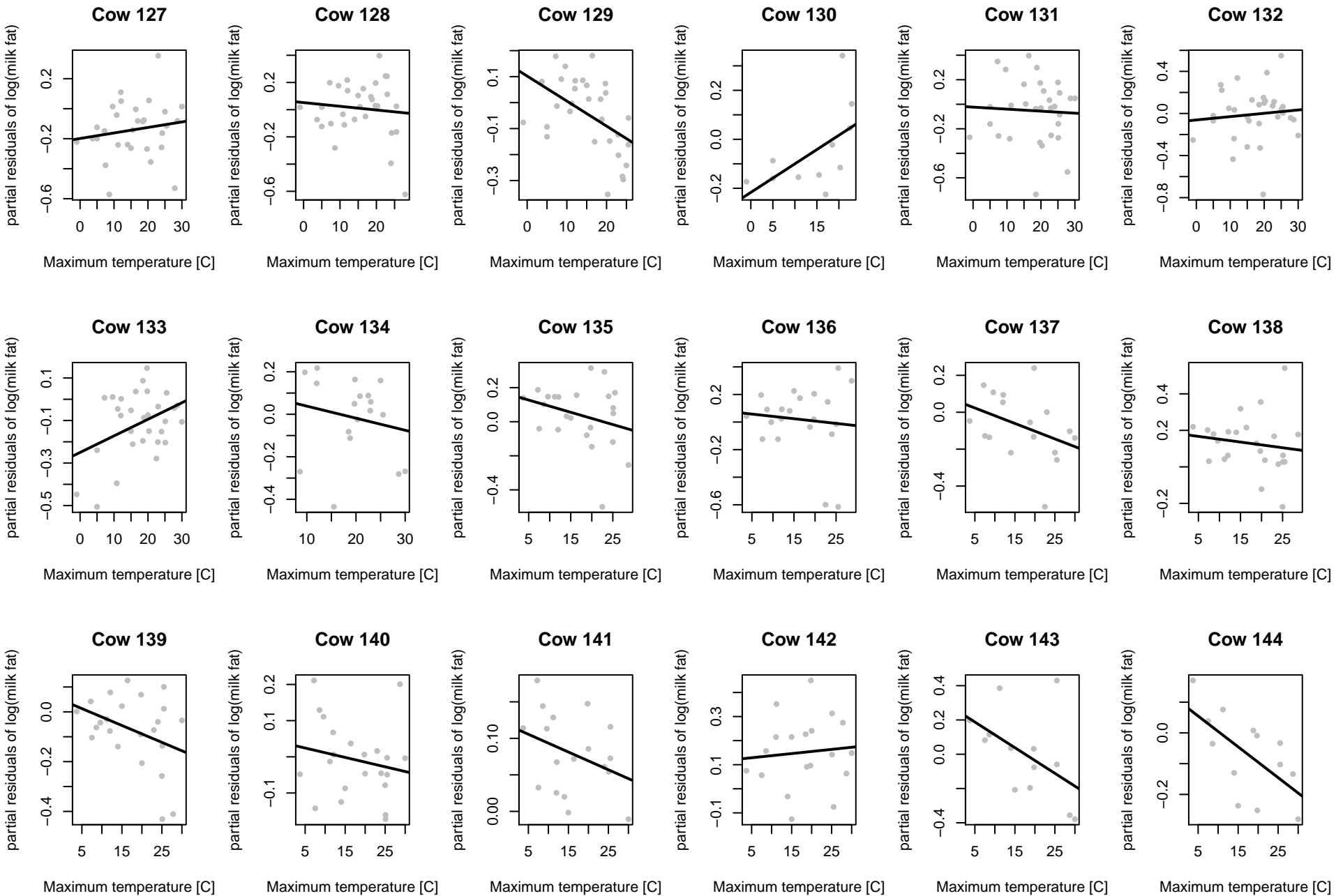

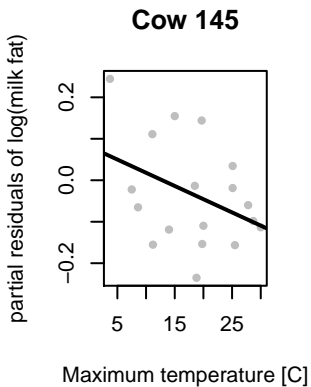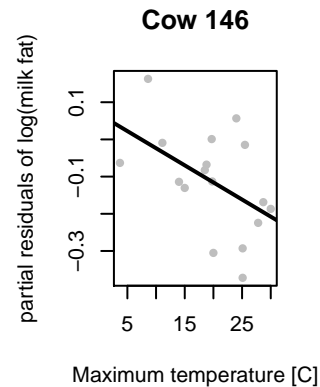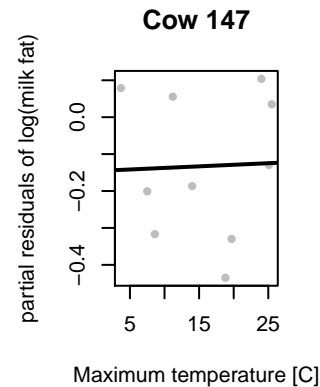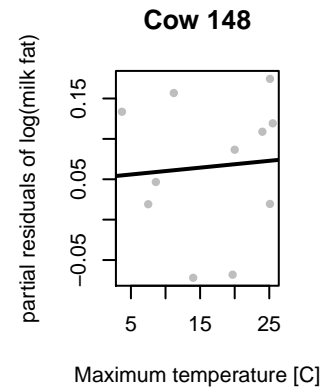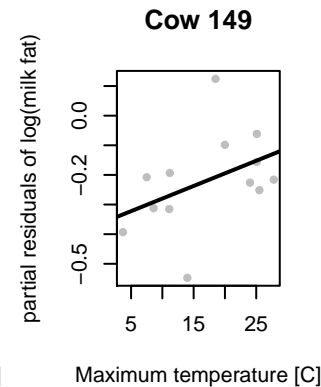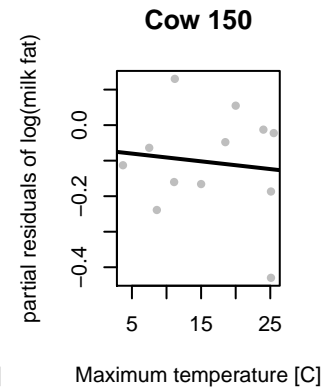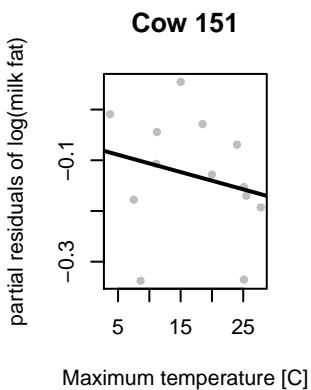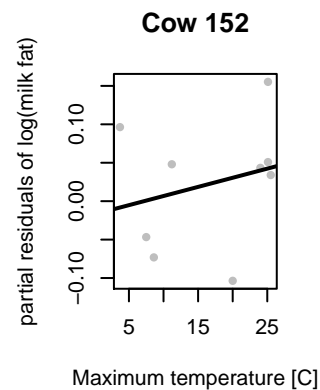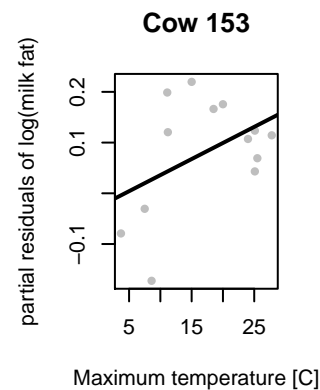

Supplement: Supplementary file 2 — Additional file 2: The partial residual plots of the milk fat, , for each cow. (PDF 76 KB) [file 40064_2013_878_MOESM2_ESM.pdf]

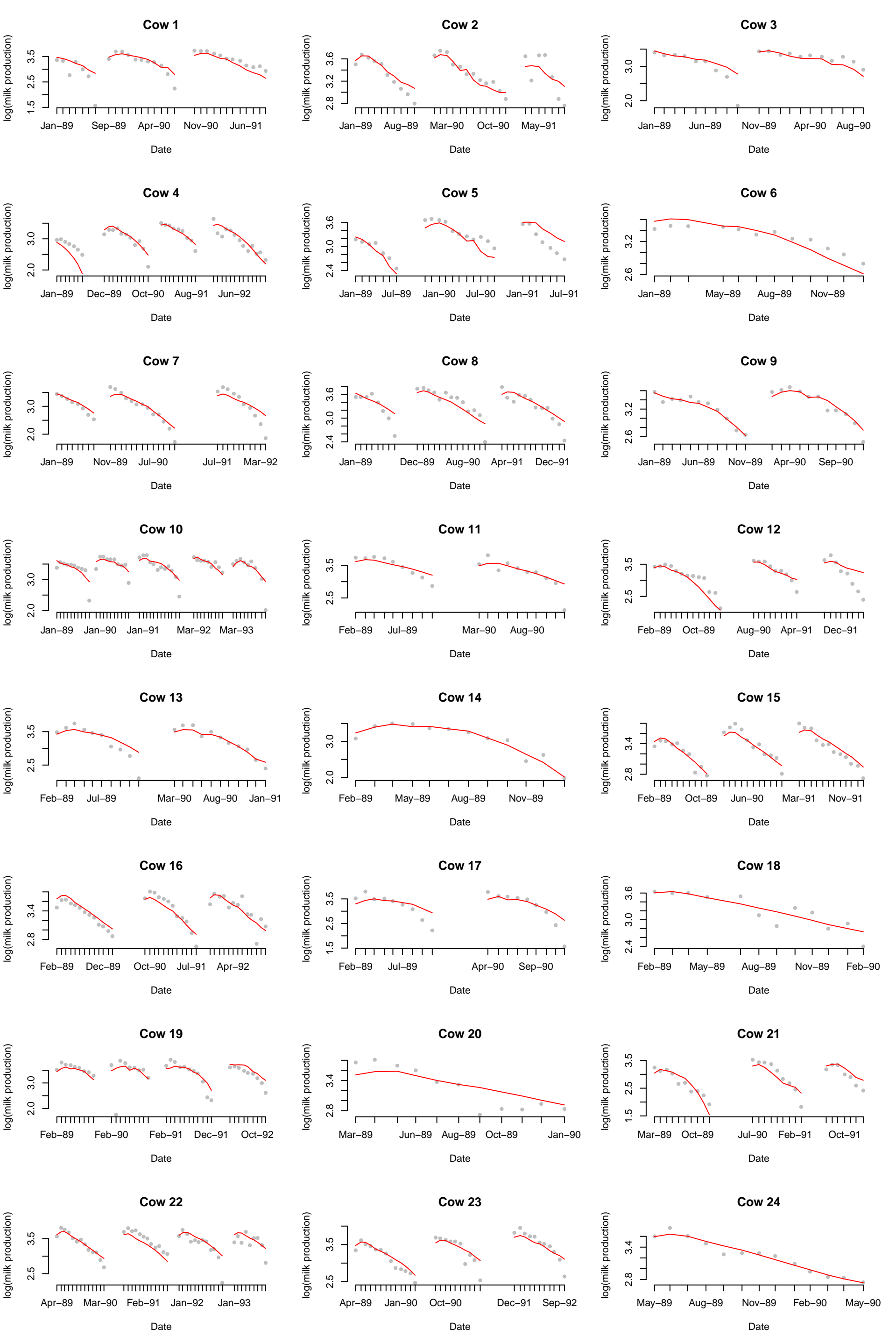

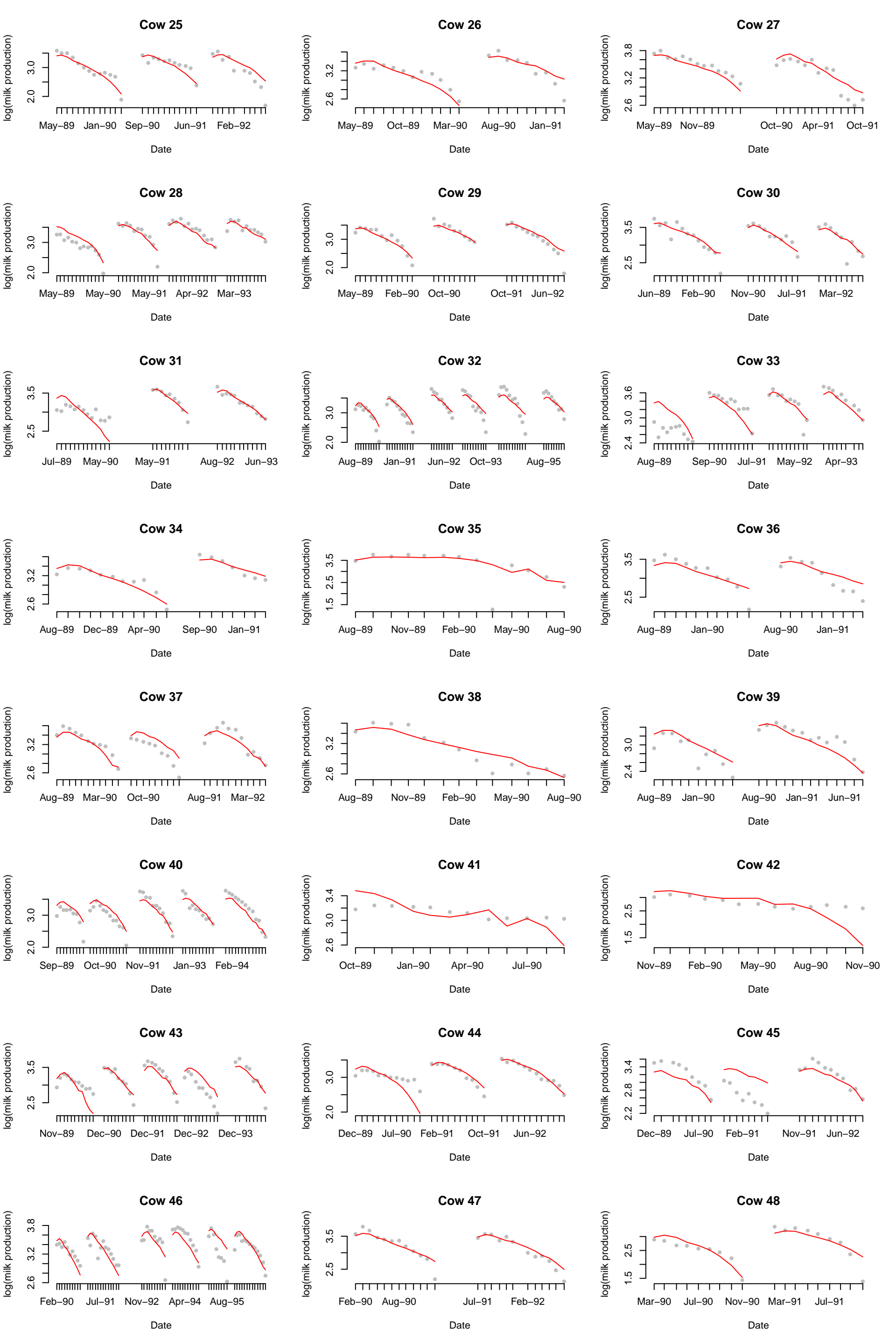

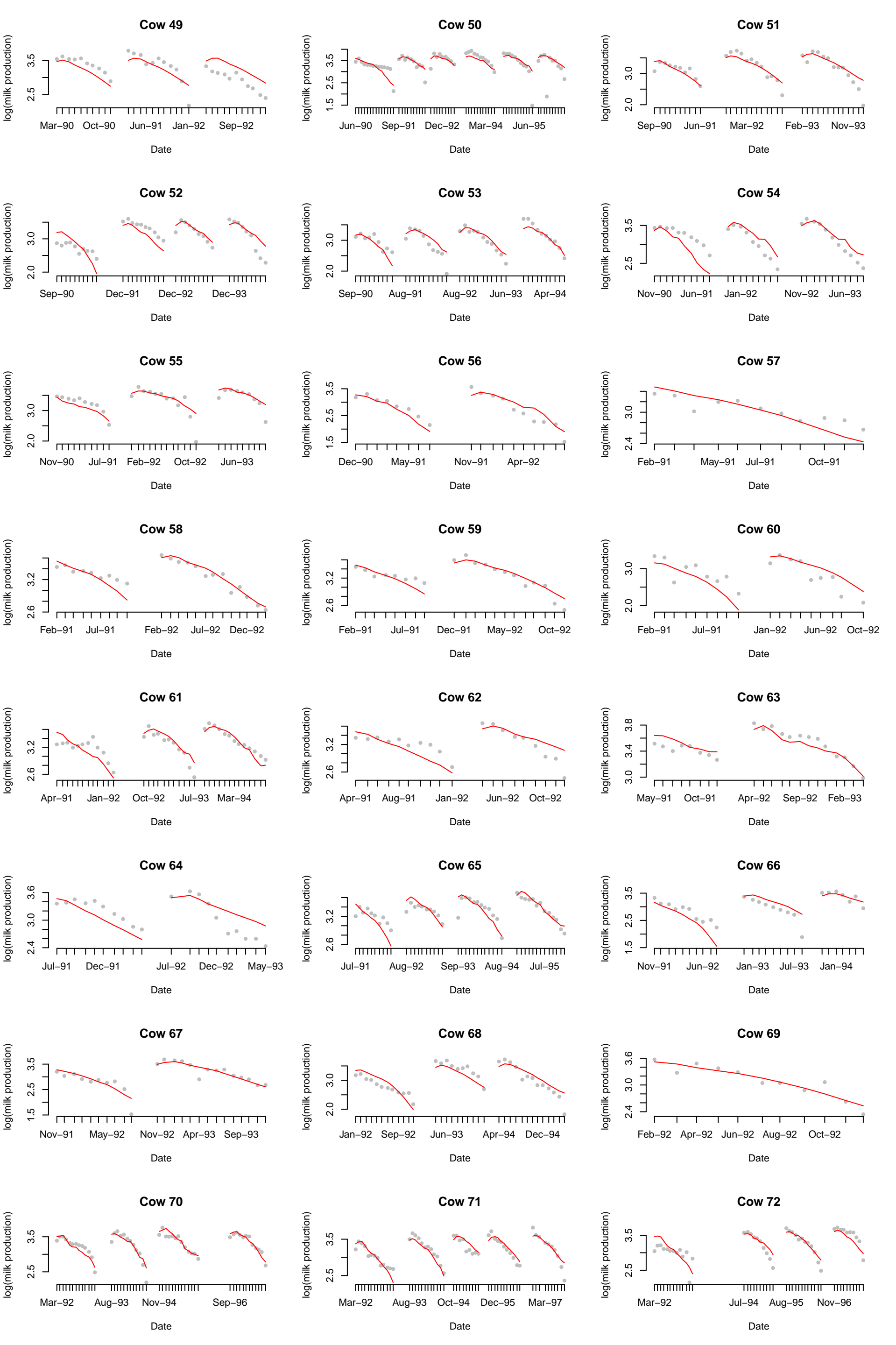

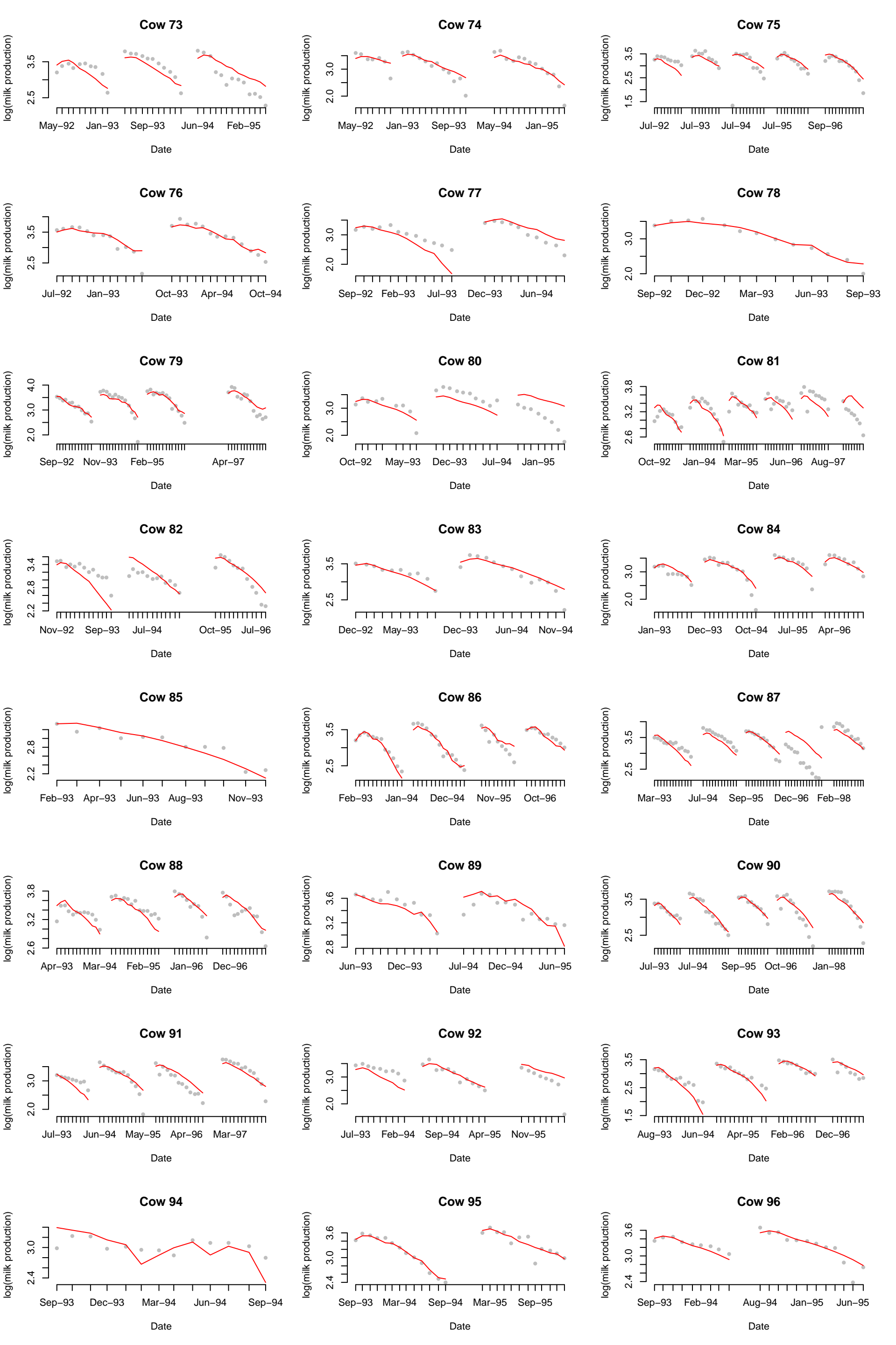

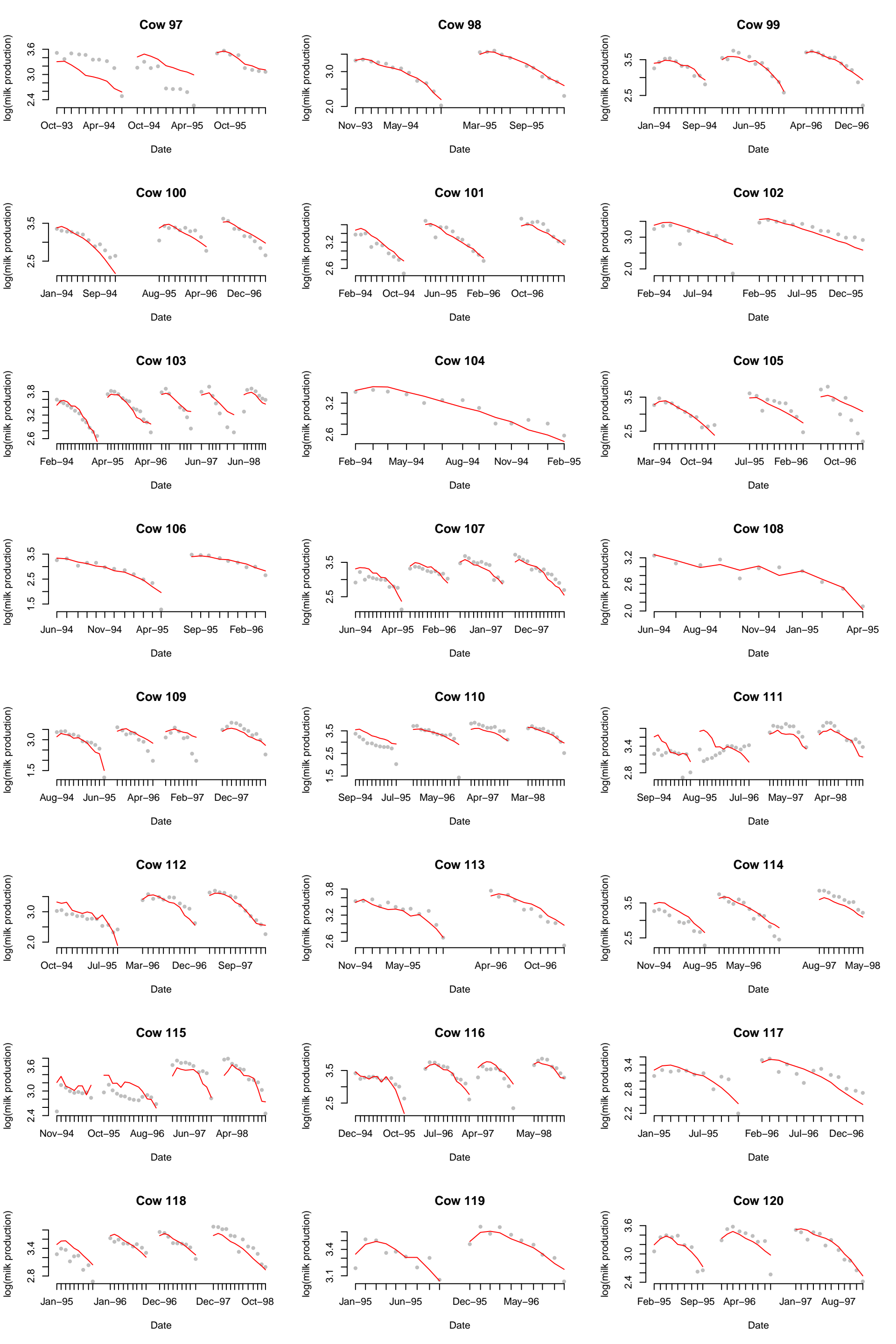

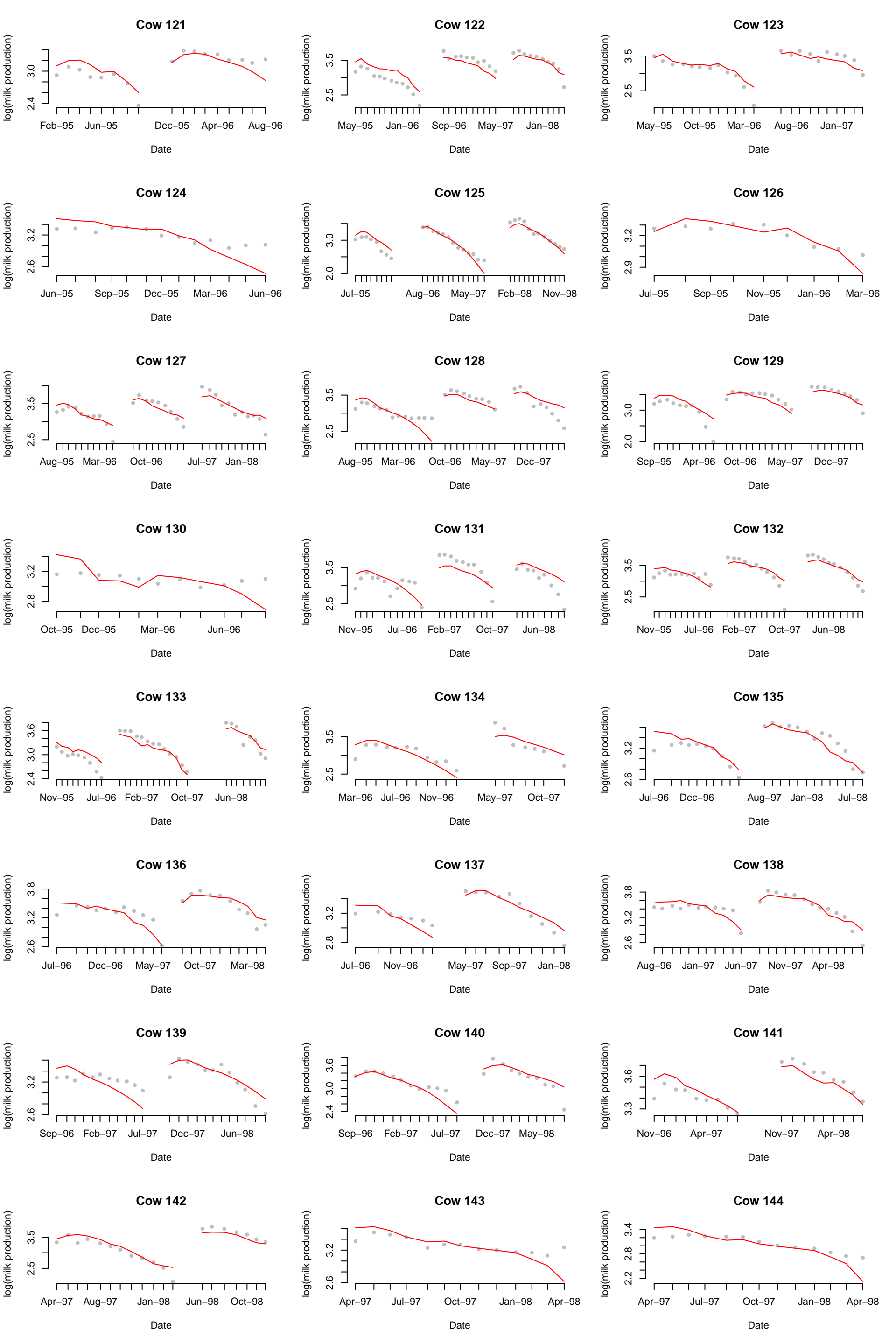

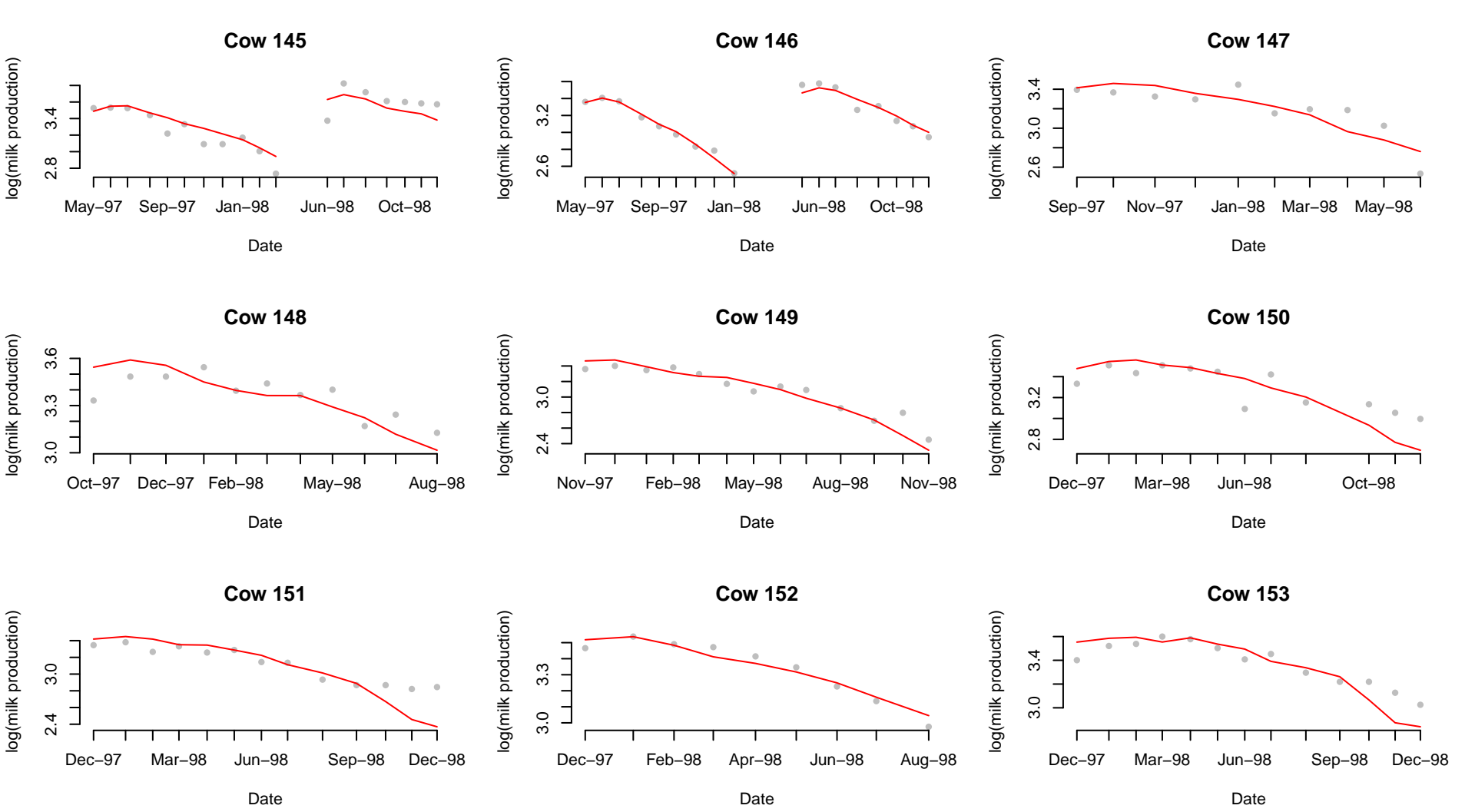

Supplement: Supplementary file 3 — Additional file 3: The fitted values of the milk production in the natural log scale for each cow, along with the observations. The superposed red line in each panel represents the fitted values. (PDF 123 KB) [file 40064_2013_878_MOESM3_ESM.pdf]

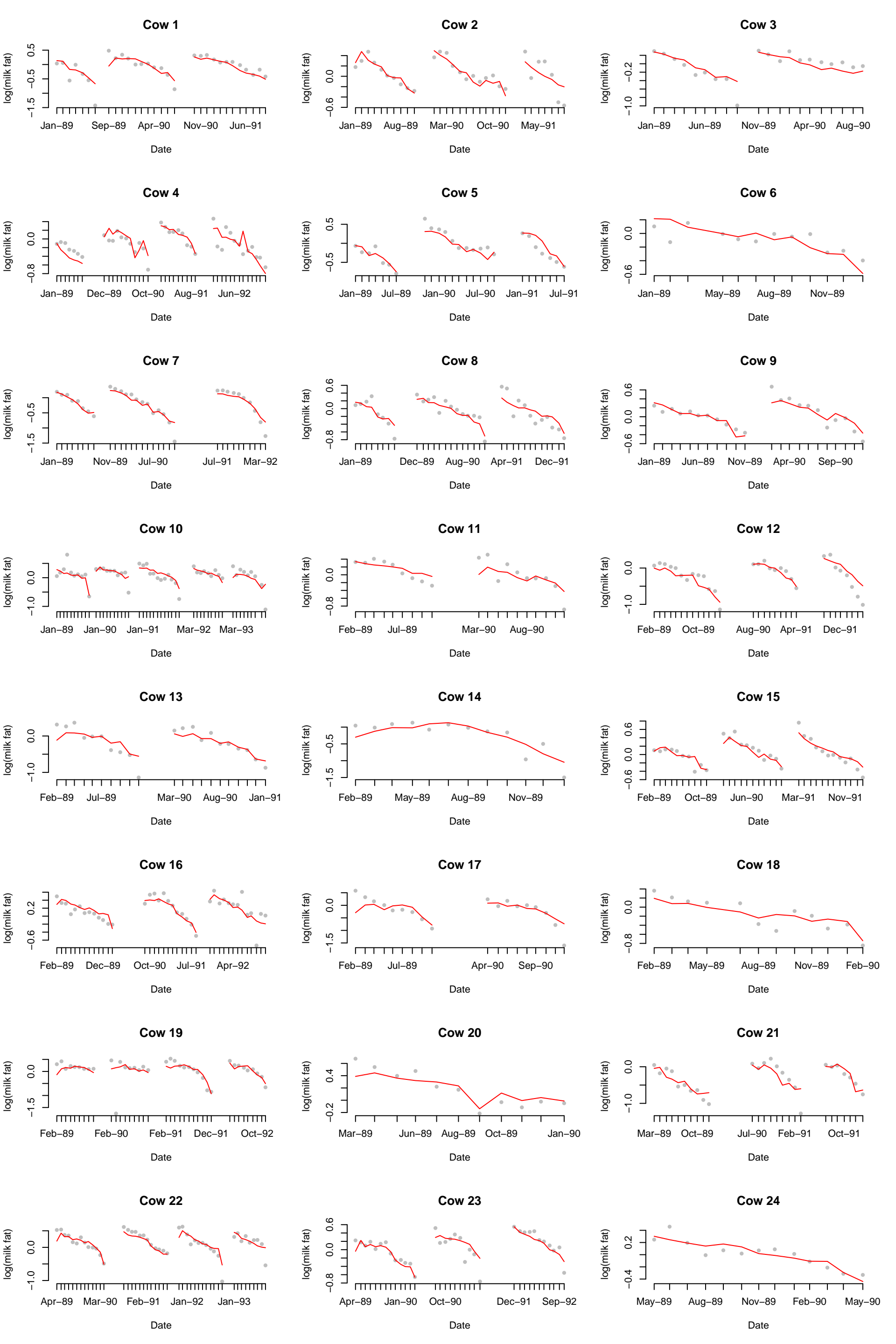

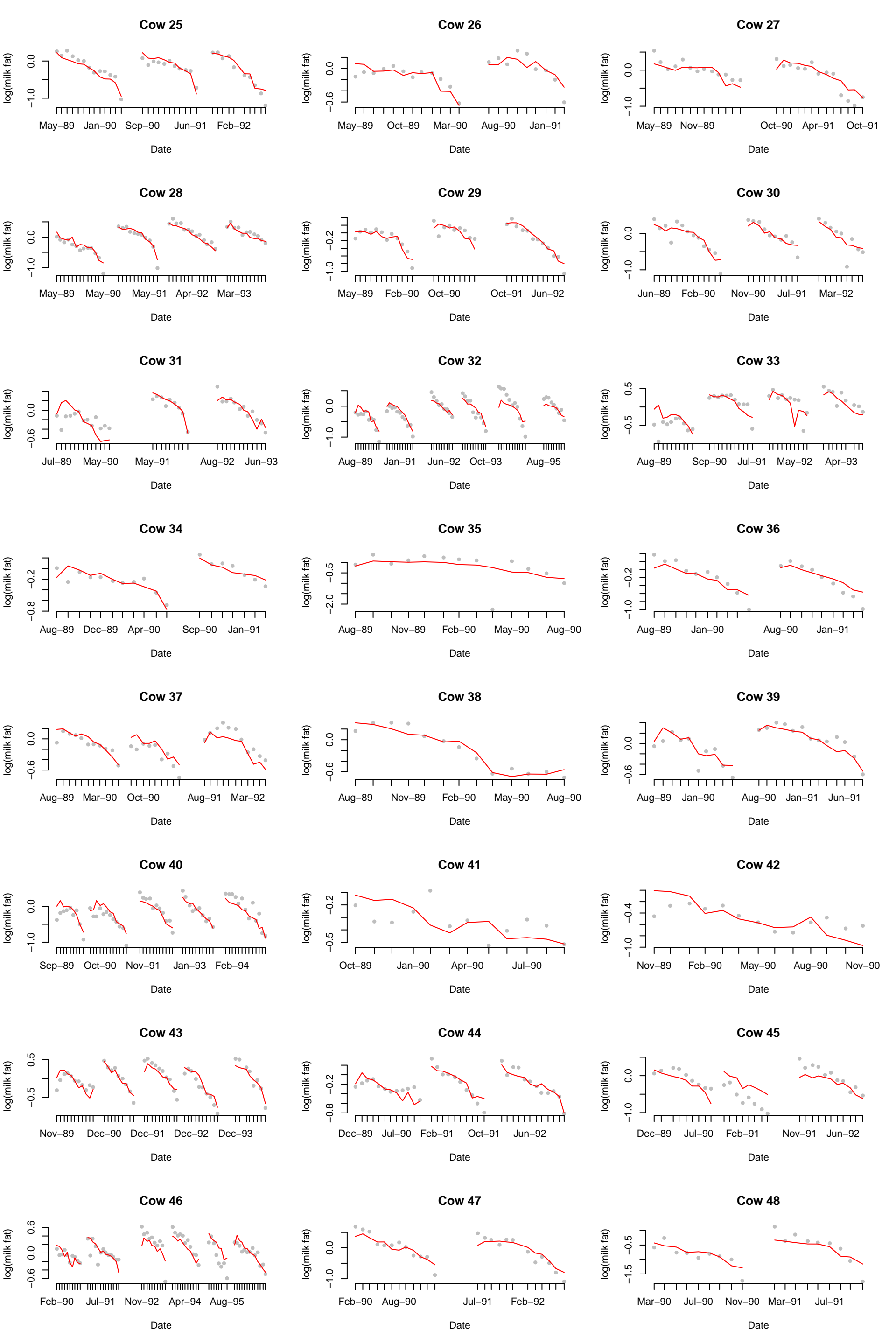

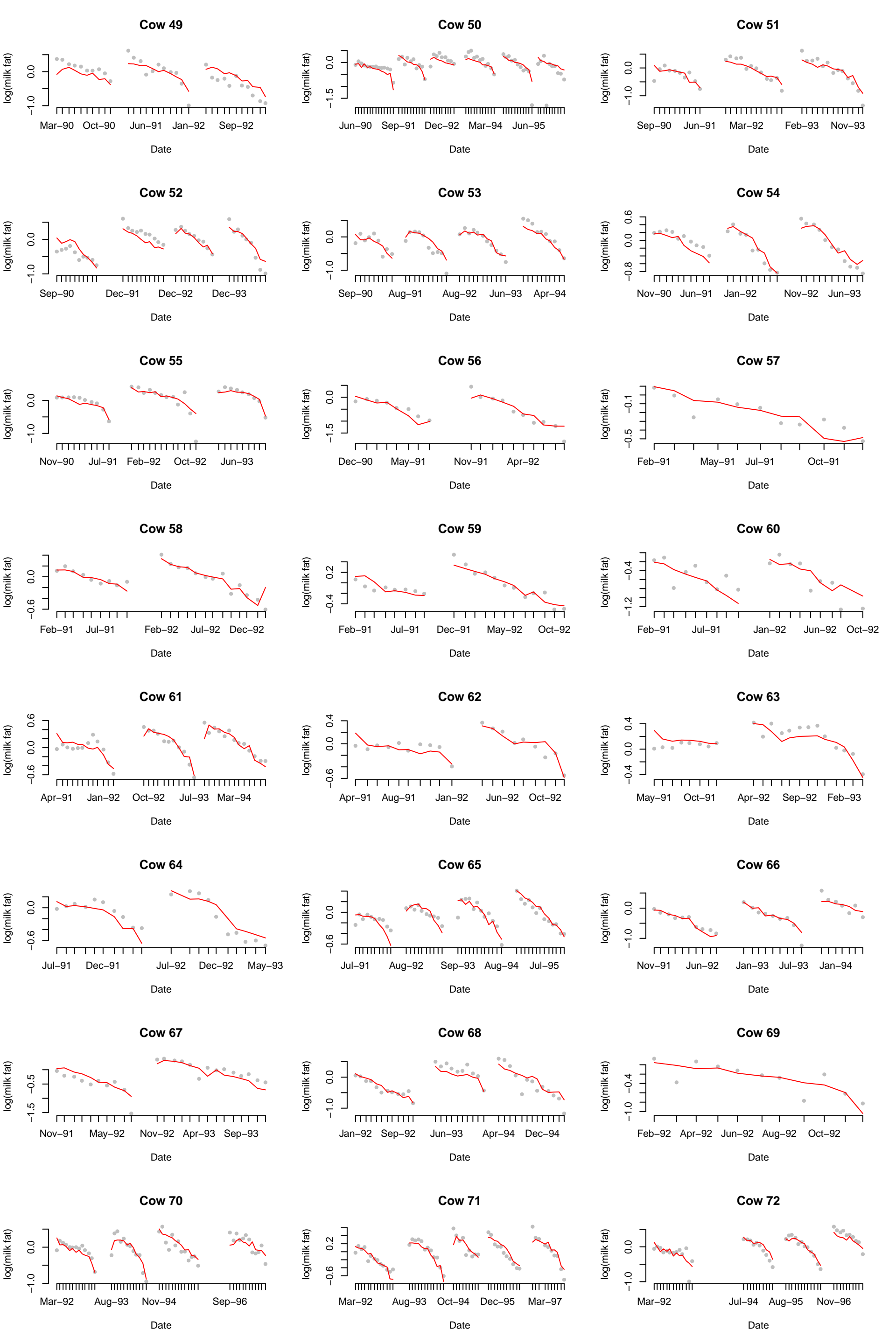

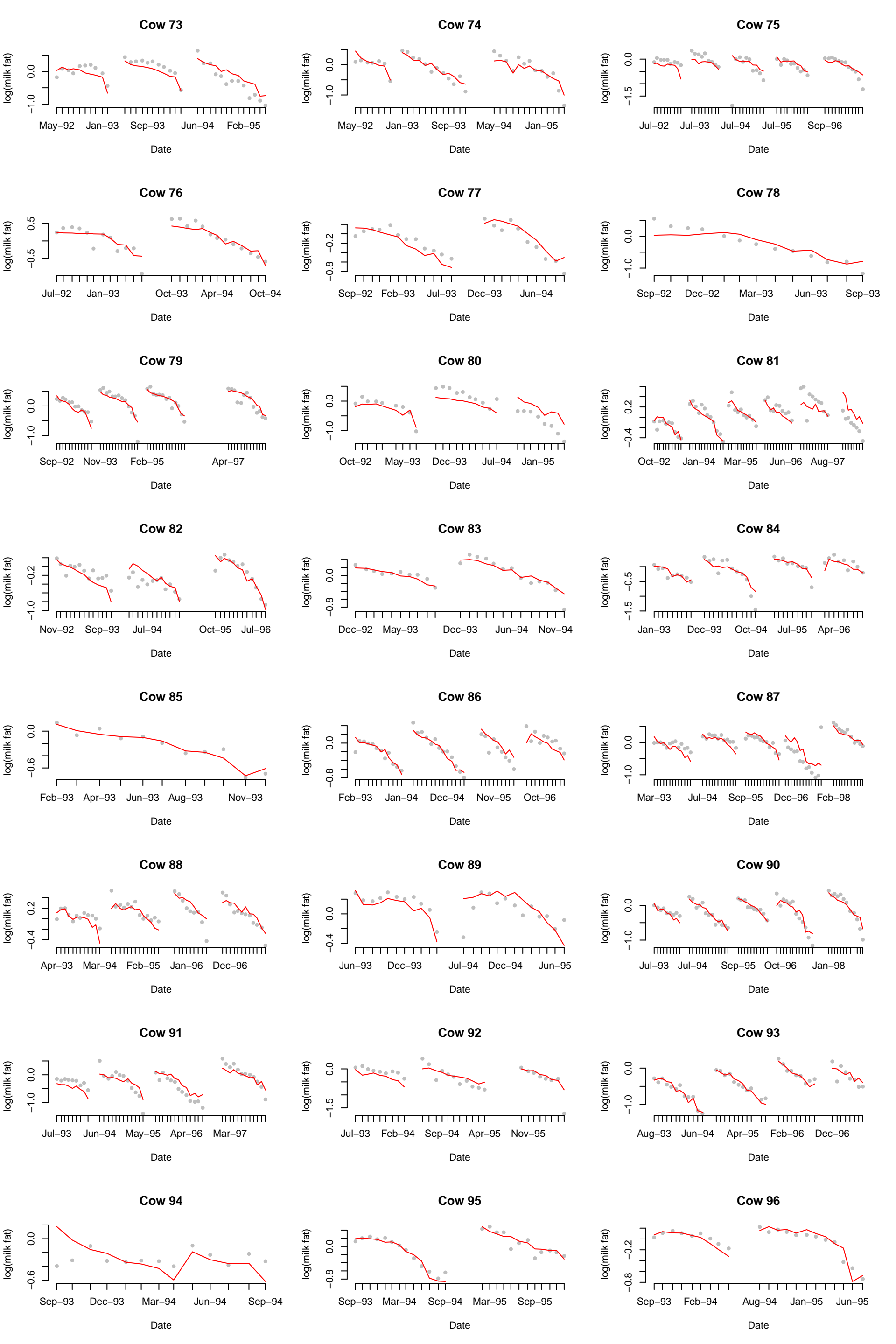

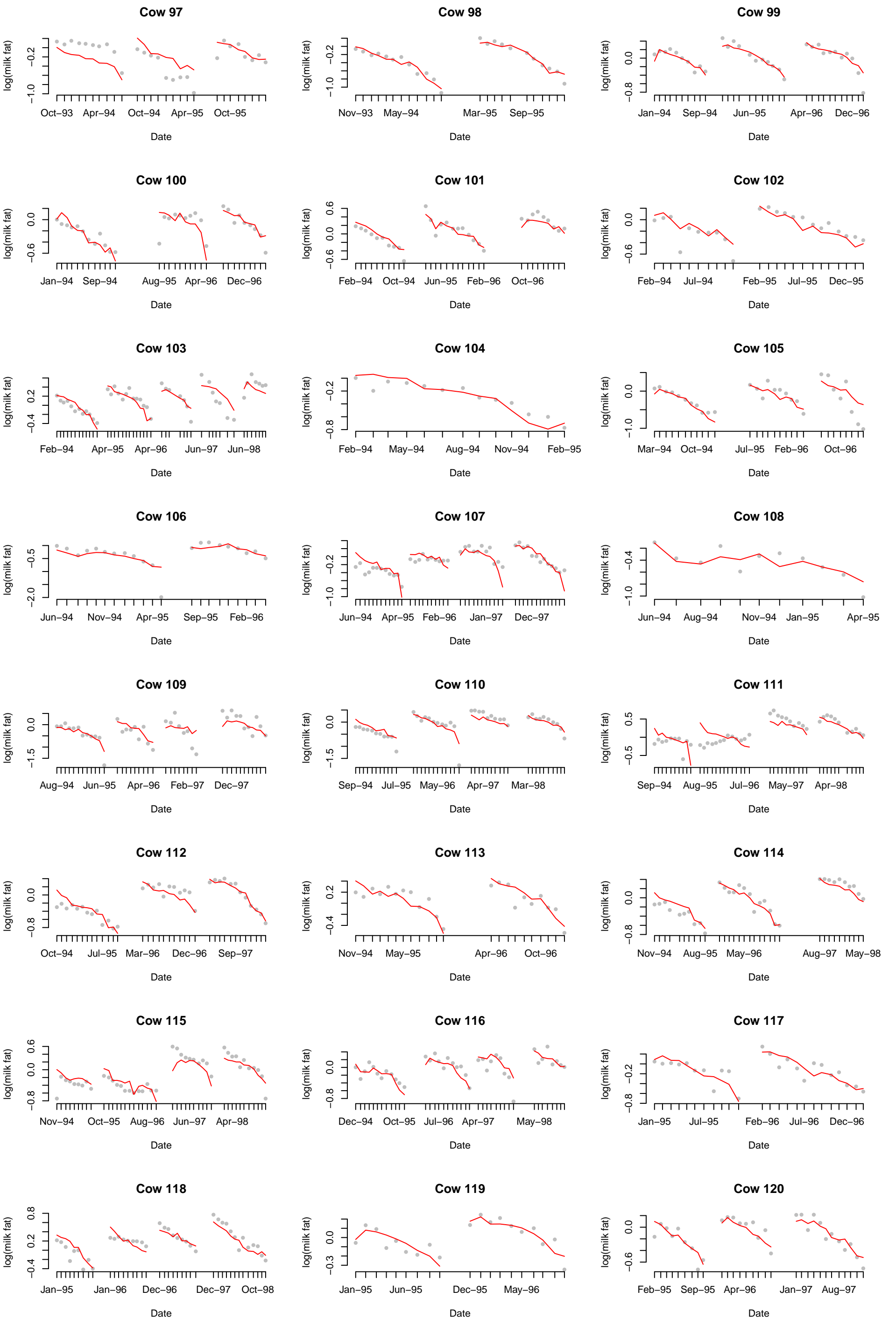

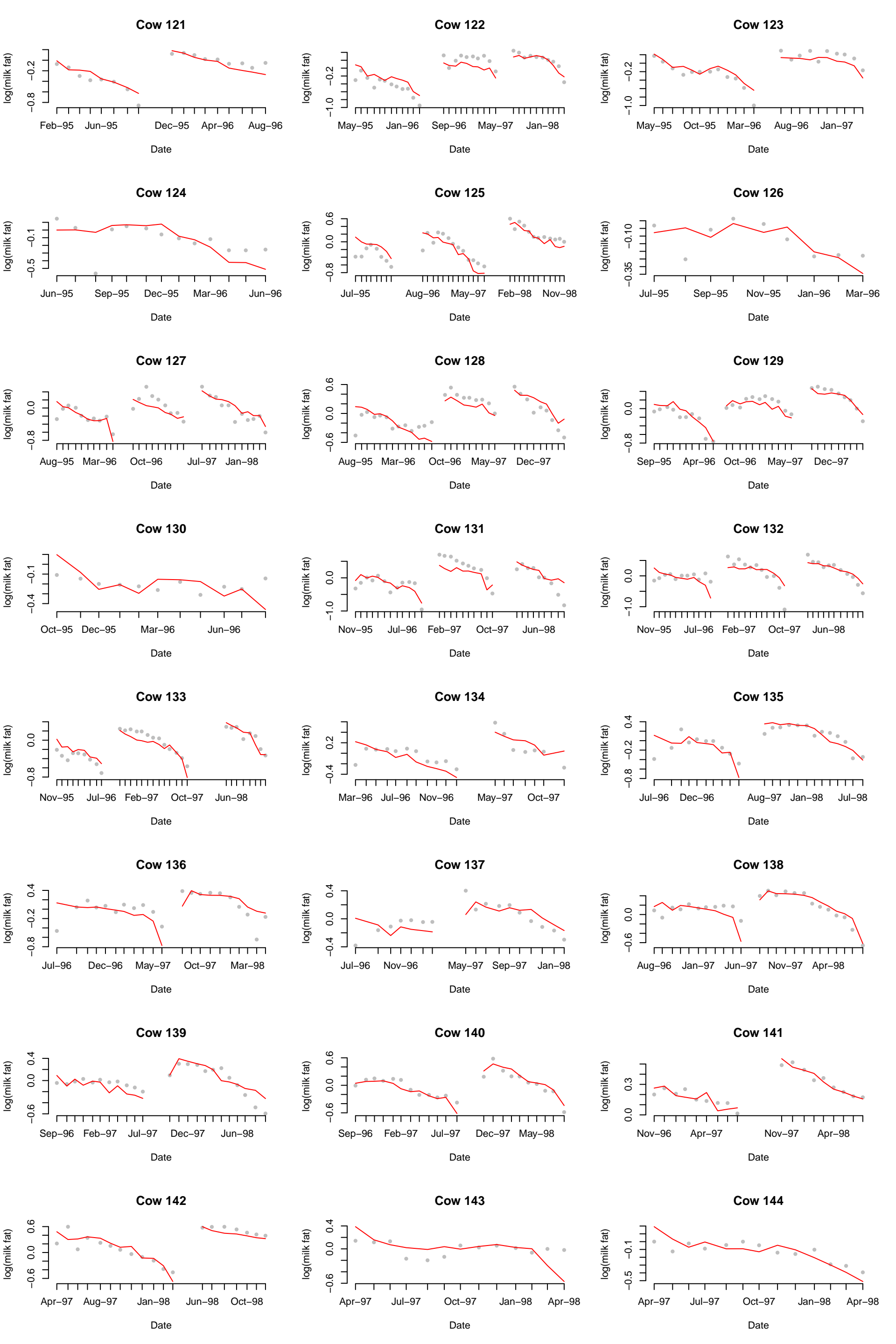

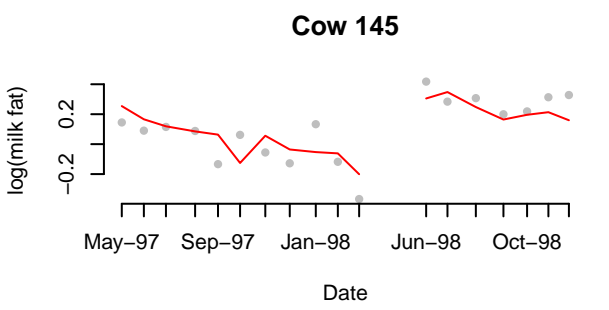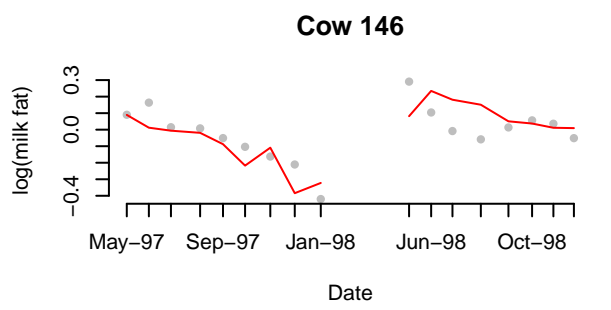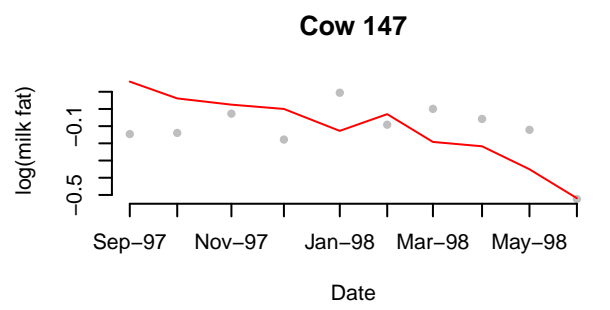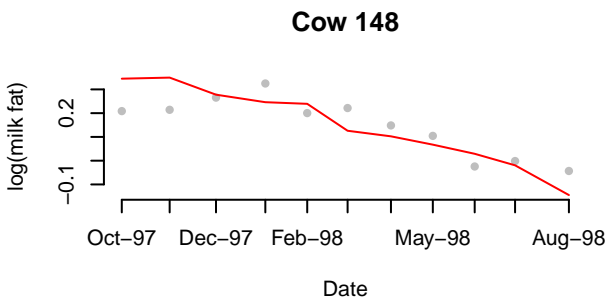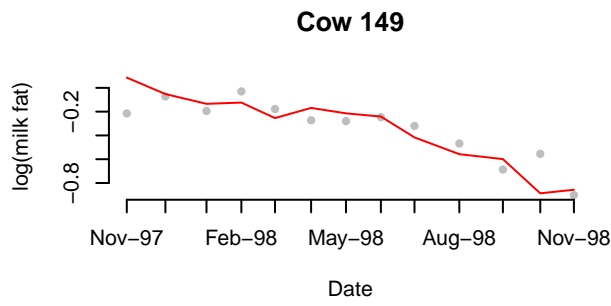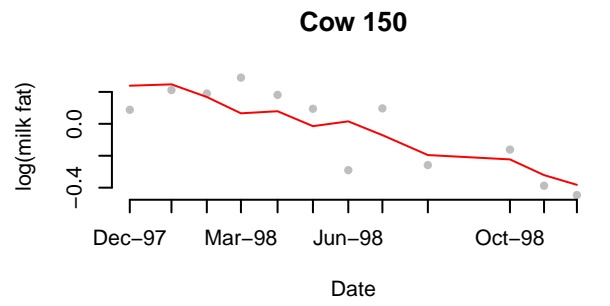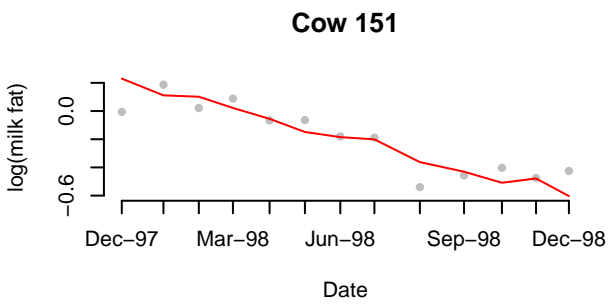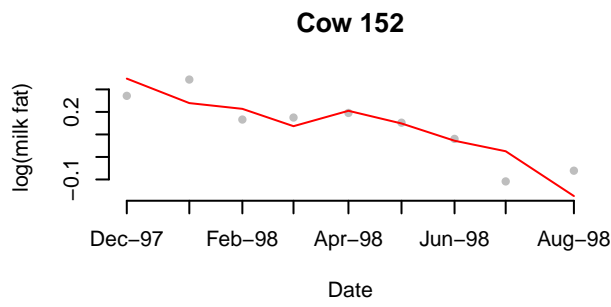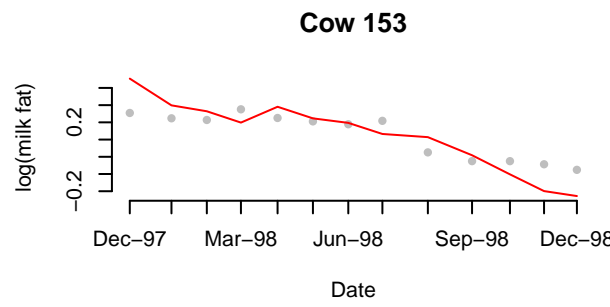

Supplement: Supplementary file 4 — Additional file 4: The fitted values of the milk fat in the natural log scale for each cow, along with the observations. The superposed red line in each panel represents the fitted values. (PDF 124 KB) [file 40064_2013_878_MOESM4_ESM.pdf]
